# New *Bis*-Alkenoic Acid Derivatives from a Marine-Derived Fungus *Fusarium solani* H915

**DOI:** 10.3390/md16120483

**Published:** 2018-12-03

**Authors:** Shun-Zhi Liu, Xia Yan, Xi-Xiang Tang, Jin-Guo Lin, Ying-Kun Qiu

**Affiliations:** 1 College of Material Engineering, Fujian Agriculture and Forestry University, Fuzhou, Fujian 350002, China; shunzhi0306@126.com; 2 Li Dak Sum Yip Yio Chin Kenneth Li Marine Biopharmaceutical Research Center, Ningbo University, Ningbo 315832, China; yanxia@nbu.edu.cn; 3 Key Laboratory of Marine Biogenetic Resources, Third Institute of Oceanography State Oceanic Administration, Xiamen 361005, China; tangxixiang@tio.org.cn; 4 Fujian Provincial Key Laboratory of Innovative Drug Target Research, School of Pharmaceutical Sciences, Xiamen University, South Xiang-An Road, Xiamen 361102, China

**Keywords:** *Fusarium solani* H915, *bis*-alkenoic acid esters, fusaridioic acid A, fusariumester A_1_, fusariumester A_2_, fusariumester B, tea pathogenic fungi inhibitory effect

## Abstract

*Fusarium solani* H915 is a fungus derived from mangrove sediments. From its ethyl acetate extract, a new alkenoic acid, fusaridioic acid A (**1**), three new *bis*-alkenoic acid esters, namely, fusariumester A_1_ (**2**), A_2_ (**3**) and B (**4**), together with three known compounds (**5**–**7**), were isolated. The structures of the new compounds were comprehensively characterized by high resolution electrospray ionization-mass spectrometry (HR-ESI-MS), 1D and 2D nuclear magnetic resonance (NMR). Additionally, the antifungal activities against tea pathogenic fungi *Pestalotiopsis theae* and *Colletotrichum gloeosporioides* were studied. The new compound, **4**, containing a β-lactone ring, exhibited moderate inhibitory activity against *P. theae*, with an MIC of 50 μg/disc. Hymeglusin (**6**), a typical β-lactone antibiotic and a terpenoid alkaloid, equisetin (**7**), exhibited potent inhibitory activities against both fungal species. The isolated compounds were evaluated for their effects on zebrafish embryo development. Equisetin clearly imparted toxic effect on zebrafish even at low concentrations. However, none of the alkenoic acid derivatives exhibited significant toxicity to zebrafish eggs, embryos, or larvae. Thus, the β-lactone containing alkenoic acid derivatives from *F. solani* H915 are low in toxicity and are potent antifungal agents against tea pathogenic fungi.

## 1. Introduction

Mangrove areas commonly experience frequent tides, strong winds, high water temperatures and exposure to strong ultraviolet radiation, while mangrove sediments typically contain high mineral composition, can readily become polluted and may be characterized as acidic, hypoxic and/or oligotrophic environments [1]. Because of the unique habitat, the discovery of new lead compounds with antibacterial activity from mangrove microorganisms has rapidly become a hot topic in the field of natural products research [2].

Tea (*Camellia sinensis* O. Kuntze) is an important economic crop in many Asian and African countries. However, tea production has been hindered by various biotic and abiotic factors. For instance, fungal diseases, especially those infecting the leaves, are among the biotic factors that cause severe damage, thereby resulting in high yield losses [3,4]. The major fungal diseases of tea leaves include blister blight (*Exobasidium vexans Massee*), grey blight (*Pestalotiopsis theae* (Sawada) Steyaert), brown blight (*Colletotrichum camelliae* Massee), sooty mold (*Capnodium theae* Boedijn) and red rust (*Cephaleuros parasiticus* Karst) [3,5,6,7,8,9].

**Figure 1:**
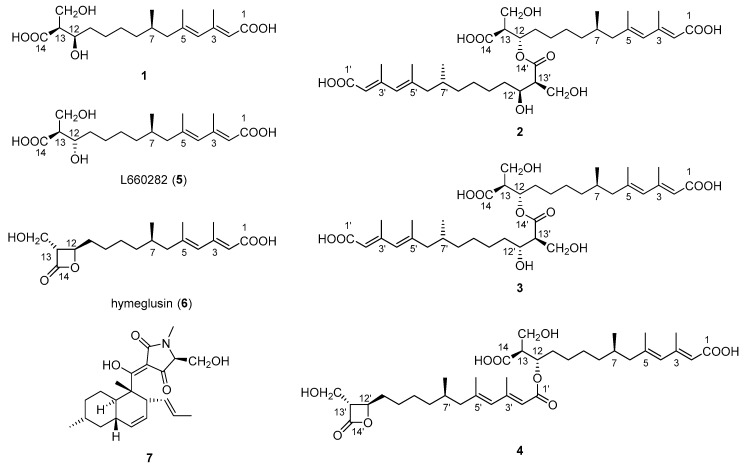
Structures of compounds **1**–**7** isolated from an extract of *Fusarium solani* H915.

To date, reports on bioactive compounds against tea pathogenic fungi are limited. Previously, we identified two new macrolactins [10,11] from *Bacillus subtilis* B5, a bacterium isolated from the 3000 m deep sea sediment of the Pacific Ocean that exhibit antifungal activity against *P. theae* and *C. gloeosporioides*. In the present study, *Fusarium solani* H915, a fungal strain that originated from the mangrove sediment of the Zhangjiangkou Mangrove National Nature Reserve, was found to possess antifungal activity. In this study, a new alkenoic acid, fusaridioic acid A (**1**), together with three new *bis*-alkenoic acid esters (Figure 1), namely, fusariumester A_1_ (**2**), fusariumester A_2_ (**3**) and fusariumester B (**4**) and three known metabolites, namely, L660282 (**5**) [12], hymeglusin (**6**) [13] and equisetin (**7**) [14], were isolated from the ethyl acetate extract of a culture of *F. solani* H915. The structures of the new compounds were comprehensively characterized by HR-ESI-MS, ^1^H NMR, ^13^C NMR and 2D-NMR. Their antifungal activities against tea pathogenic fungi *P. theae* and *C. gloeosporioides* were studied. Equisetin and the alkenoic acid derivatives containing a β-lactone ring exhibited inhibitory activities against the fungi. However, equisetin exhibited strong anti-proliferative effects on zebrafish embryos and larvae. The alkenoic acid derivatives with β-lactone rings from *F. solani* H915 are low in toxicity and thus may be potentially used as antifungal agents against tea pathogenic fungi.

## 2. Results

### 2.1. Structural Identification of New Compounds

**Figure 2:**
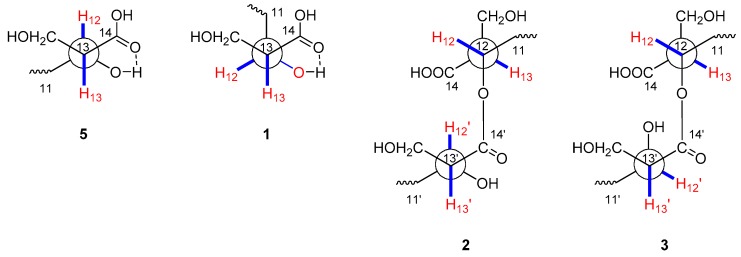
Key preferential conformations of **1**–**3** and **5**.

**Figure 3:**
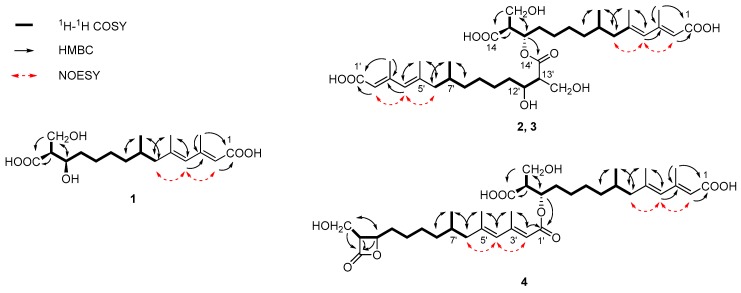
Key ^1^H–^1^H COSY, HMBC and NOESY correlations of **1**–**4**.

Fusaridioic acid A (**1**) was isolated as a white amorphous powder. The infrared (IR) spectrum of **1** indicated the presence of free and conjugated carboxylic acid carbonyl signals at 1734 cm^−1^ and 1683 cm^−1^, respectively. The UV maximum absorption wavelengths at *λ*_max_ (log *ε*), 233 (3.58) nm and 266 (3.80) nm, belong to an unconjugated carbonyl and another conjugated carbonyl, respectively. Its molecular formula of C_18_H_30_O_6_, which gave four unsaturation degrees, was established by the HR-ESI-MS at 341.1968 [M − H^+^]^−^ (calcd. for 341.1964 C_18_H_29_O_6_) and by the data of 1D-NMR. In the low field of ^1^H NMR, two olefinic proton signals were observed with br. s peaks at δ_H_ 5.50 (H-2) and 5.67 (H-4). In the *sp*^3^ region of the ^1^H NMR spectrum, two methyl groups at δ_H_ 2.09 (d, *J* = 1.1 Hz, 3-CH_3_) and 1.70 (d, *J* = 1.1 Hz, 5-CH_3_) were considered linked to quaternary olefinic carbons. Another methyl at δ_H_ 0.73 (d, *J* = 6.6 Hz, 7-CH_3_) is connected to a methylene. In the *sp*^2^ region of ^13^C NMR a carboxyl signal emerged at δ_C_ 174.9 (C-14), whereas another one (which is conjugated with the double-bond system) presented at δ_C_ 168.1 (C-1). The *sp*^3^ high-field region of the ^1^H NMR spectrum showed the existence of three methyl proton signals. Two methyl groups with *J* values of 1.1 Hz at δ_H_ 2.09 (3-CH_3_) and 1.70 (5-CH_3_) were considered linked to quaternary olefinic carbons. The other methyl at δ_H_ 0.73 (d, *J* = 6.6 Hz, 7-CH_3_) is connected to a methylene. In addition, a methine and a methylene, bound to oxygen atoms, could be found in the ^13^C NMR and distortionless enhancement by polarization transfer (DEPT) spectra at δ_C_ 69.3 (C-12) and 55.4 (13-**C**H_2_OH). With the aid of ^1^H-^1^H homonuclear chemical shift correlation spectroscopy (COSY) spectra, the proton signal at δ_H_ 3.55 (m) was attributed to H-12. The two dd peaks at δ_H_ 3.51 (*J* = 10.5, 8.4 Hz) and 3.46 (*J* = 10.4, 5.4 Hz), which form a typical ABX coupling system with H-13, were assigned to the two protons at 13-CH_2_OH. Elucidation of heteronuclear single quantum coherence (HSQC), ^1^H-^1^H COSY and ^1^H detected heteronuclear multiple bond correlation (HMBC) spectra, led to the planar structure (Figure 1), which is almost identical to that of L-660282 (**5**), a compound isolated from a culture of *Cephalosporium* sp. [12]. Indeed, most of the 1D NMR spectral data of **1** approached those of L-660282 (**5**) (Table 1 and Table 2). The ^13^C NMR signal differences between **1** and **5** are found at positions 11, 12, 13, 14 and 13-CH_2_OH (δ_C_ 35.0, 69.3, 55.4, 174.9 and 60.1 in **1**; and δ_C_ 35.7, 69.5, 56.0, 175.2 and 61.3 in **5**), revealing that **1** is an epimer of **5** at 12-OH. In both compounds, the C-14 carbonyl oxygen forms a hydrogen bond to 12-OH. At the same time, the 14-carboxyl hydroxyl group can form a hydrogen bond with the 13-CH_2_OH group. Both those H-bonding interactions will lead to six-membered rings for both isomers. In compound **5**, H-12 and H-13 are in a trans-coplanar position, inducing an H12-H13 coupling constant of 8.3 Hz (due to the presence of such electron withdrawing groups as hydroxyl and carbonyl, the coupling constant is slightly lower than the Karplus equation prediction coupling value). However, the bond angle between H-12 and H-13 in **1** is about 60°, leading to a reduction of the *J*_H-12, 13_ value to 5.7 Hz (Figure 2). Thus, the coupling splittings of H-13 in **5** present a td peak with *J* values of 8.5 Hz × 2 and 4.6 Hz × 1, whereas those in **1** led to a dt peak with *J* values of 8.3 Hz × 1 and 5.7 Hz × 1. The structural connection of **1** was further confirmed by the HSQC, ^1^H-^1^H COSY and HMBC spectra (Figure 3). The configuration of the two double bonds was revealed by the nuclear overhauser effect spectroscopy (NOESY) spectrum (Supplementary Materials Figures S1–S8).

The absolute configuration of C-7, C-12 and C-13 in **1** was revealed by alkaline hydrolysis of hymeglusin (**6**), a typical β-lactone antibiotic previously isolated from a culture of *Scopulariopsis* sp. F-244 [13]. Alkaline hydrolysis of hymeglusin (**6**), which mainly experienced a bi-molecular substitution nucleophilic (SN2) reaction process, yielded a single product, whose thin layer chromatography (TLC) retardation factor (Rf) value, high performance liquid chromatography (HPLC) retention time and optical rotation ([α]${}_{D}^{25}$) value were close to those of compound **1**, indicating that the absolute configuration of **1** should be 7*R,* 12*R,* 13*S*.

Fusariumester A_1_ (**2**) was isolated as colorless viscous oil, whose [α]${}_{D}^{25}$ value was close to 0° (*c* = 0.1, CH_3_OH). Its molecular formula of C_36_H_58_O_11_, which gave eight unsaturation degrees, was established by the HRESIMS at 665.3883 [M − H^+^]^−^ (calcd. for 665.3901 C_36_H_57_O_11_) and by the data of 1D NMR. The IR spectrum showed a wide and strong adsorption signal band at 1695 cm^−1^, caused by overlapping of multiple carbonyl signals. Absorption at 1631 cm^−1^ belongs to the conjugated C=C system. The ^1^H and ^13^C NMR data of **2**, whose signals emerged in duplicate pairs, indicated the presence of two similar structural units. The NMR data of each unit were close to those of **5**, indicating that **2** could be a dimer of **5**. In detail, four olefinic proton signals were found in the low field region of the ^1^H NMR spectrum of **2**, at δ_H_ 5.70 (1H, br.s, H-4), 5.71 (1H, br.s, H-4′), 5.60 (1H, br.s, H-2) and 5.56 (1H, br.s, H-2′). With the aid of ^13^C NMR, DEPT, HSQC and HMBC spectra, their corresponding carbon signals belonging to two pairs of conjugated dienes could be assigned. Three pairs of methyl signals were also found in the high-field region of the ^1^H and ^13^C NMR spectra. All these pairs of signals, together with a pair of carboxyls [δ_C_ 168.3 (C-1) and 168.2 (C-1′)] and some other *sp*^3^ carbons (C-6, 7, 8, 9 and C-6′, 7′, 8′, 9′), were paired very well, with almost identical chemical shifts. However, the chemical shifts of the ^1^H and ^13^C atoms at positions 11-14 and 11′-14′ were different, indicating that the two structural units of **5** are linked with each other at these positions. In the ^13^C NMR spectrum, δ_C_ 174.4 (C-14) differed from 173.3 (C-14′); δ_C_ 53.3 (C-13) differed from 56.2 (C-13′); δ_C_ 71.6 (C-12) differed from 69.3 (C-12′); δ_C_ 32.6 (C-11) differed from 35.8 (C-11′) and δ_C_ 60.6 (13-CH_2_OH) differed from 61.5 (13′-CH_2_OH). Compared with those of compound **5**, the low-field shift of C-12 and high-field shifts of C-13, C-11 and C-14′ revealed esterification between the 12-hydroxyl and 14′-carboxyl. This finding was further confirmed by the HMBC correlation between H-12 [δ_H_ 5.00 (1H, m)] and C-14′ (δ_C_ 173.3), as well as the low-field shifting of H-12. The relative position between H-12 and 13-CH_2_OH and that between H-12′ and 13′-CH_2_OH were both deduced as trans coplanar positions, by the coupling constants and the td peak splitting of H-13 and H-13′ in ^1^H NMR (Figure 2). Alkaline hydrolysis of **2** yielded a single product, compound **5**, indicating that compound **2** is an esterified product of two molecules of compound **5**. The full assignment of ^1^H (Table 1) and ^13^C NMR data (Table 2) was deduced by careful elucidation of HSQC, ^1^H-^1^H COSY, HMBC and NOESY spectra (Figure 3, Supplementary Materials Figures S9–S17). The absolute configurations of C-7, 7′, C-12, 12′ and C-13, 13′ in **2** were identical to those in compound **5**, because the TLC Rf value, HPLC retention time and optical rotation value of the alkaline hydrolysis of **2** were almost identical to those of **5** (Supplementary Materials Figure S35). Considering that compounds **1** and **5** are a pair of epimers that differ at C-12 and their similar biosynthetic pathway, the absolute configuration of **2** should be 7*R*, 12*S*, 13*S* and 7′*R*, 12′*S*, 13′*S*.

Fusariumester A_2_ (**3**) was isolated as colorless viscous oil. The ^1^H and ^13^C NMR data of **3** were almost identical with those of compound **2** except for the protons and carbons around C-13′. In detail, compared with those of **2**, chemical shift of H-12′ in **3** is low-field shifted from δ_H_ 3.50 to δ_H_ 3.62; that of H-13′ is low-field shifted from δ_H_ 2.44 to δ_H_ 2.50; and that of 13′-CH_2_OH is high-field shifted from δ_H_ 3.76 and 3.61 to δ_H_ 3.61 and 3.56. In the ^13^C NMR of **3**, the signals of C-13′, 14′ and 13′-**C**H_2_OH are high-field shifted about 0.5–1.5 ppm. The structure of **3** was deduced to be an epimer of compound **2** at C-12′ and C-13′. This deduction is confirmed by the coupling constants and the dt peak splitting of H-13′ in ^1^H NMR (Figure 2). Alkaline hydrolysis of **3** yielded a pair of products, compounds **1** and **5**, indicating that compound **3** is an esterified product of compound **1** and **5**. The absolute configuration of **3** is 7*R*, 12*R*, 13*S* and 7′*R*, 12′*S*, 13′*S*, which was confirmed by comparing the TLC Rf value, HPLC retention time and optical rotation value of the alkaline hydrolysis of **3** with those of compounds **1** and **5** (Supplementary Materials Figure S35).

Fusariumester B (**4**) is a colorless viscous oil. Its molecular formula of C_36_H_56_O_10_ established by the HRESIMS quasi-molecular ion peak at *m/z* 647.3814 [M − H^+^]^−^ (calcd. for 647.3873 C_36_H_55_O_10_), yields nine unsaturation degrees. The molecular weight of **4** is lower than those of **2** and **3** by 18 Da, indicating that **4** could be the closed-ring product of **2** or **3**. The IR carbonyl absorption at 1822 cm^−1^ indicated the presence of a β-lactone group. Carbonyl signals at 1715 and 1698 cm^−1^ were attributed to other carbonyl groups. Most of the ^1^H, ^13^C NMR and DEPT data of **4** were similar with those of **2** except the carbon signals assigned to C-1′, 12′, 13′, 14′ and 13′-CH_2_OH. Indeed, the NMR data of subunit C-1′–C-14′ are close to those of hymeglusin (**6**), indicating the presence of β-lactone in **4**. The β-lactone structure leads to the chemical shift changing of the atoms at positions 12′, 13′, 14″ and 13′-CH_2_OH. On the other hand, compared with the ^13^C NMR data of hymeglusin reported in Reference [, the chemical shift of C-1′ was high-field shifted from 172.0 to 166.1 and that of C-2′ was low-field shifted from 116.7 to 118.6, indicating esterification of the 1′-carboxyl. Based on the correlation between H-12 [δ_H_ 5.05 (1H, q-like, *J* = 6.5 Hz)] and C-1′ (δ_C_ 166.1) in HMBC spectrum, 1′-carboxyl is confirmed to be esterified with 12-hydroxyl. Alkaline hydrolysis of **4** also yielded a pair of products, namely, compounds **1** and **5**. The open-ring diacid structure unit led to the yield of compound **1**, while the β-lactone structure unit underwent an open-ring SN2 alkaline hydrolysis ring opening to yield compound **5** as one of the final products (Supplementary Materials Figure S35). Base on the above findings, the absolute configuration of **4** is 7*R*, 12*S*, 13*S* and 7′*R*, 12′*R*, 13′*R*.

### 2.2. Antifungal Activity

*P. theae* (HQ832793) and *C. gloeosporioides* (HQ832797) were isolated from foliar lesions of tea leaf and their pathogenicity to tea leaf were verified both in vitro and in vivo.

The antifungal activity of the isolated compounds was evaluated by the paper disc inhibition assay and the minimum inhibitory concentration (MIC) was determined by the paper disc dilution method as described previously [15]. The new compound **4**, containing a β-lactone ring, exhibited moderate inhibitory activities with MIC of 50 μg/disc for *P. theae*, while showing little activity with *C. gloeosporioides*. Hymeglusin (**6**), a typical β-lactone antibiotic and equisetin (**7**), a terpenoid alkaloid, exhibited potent inhibitory activities against both fungi with an MIC value of 25 μg/disc.

### 2.3. Toxicity Evaluation

**Figure 4:**
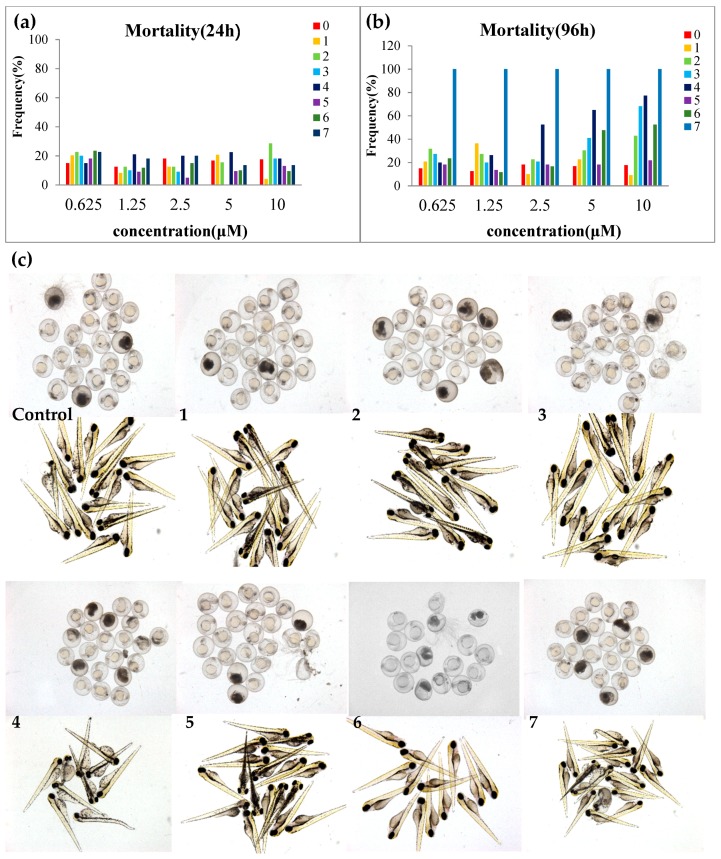
Toxicity evaluation of compounds **1**–**7** on zebrafish model. (**a**) Mortality of zebrafish embryo at 24 h. (**b**) Mortality of zebrafish larva at 96 h. (**c**) Photograph of zebrafish embryo (24 h) and zebrafish larva (96 h). The final concentration of compounds **1**–**6** was 10 μM and that of compound **7** was 0.31 μM.

All the isolated compounds were evaluated for their anti-proliferative effects on zebrafish embryos (Figure 4). Equisetin (**7**) showed strong anti-proliferative effects, leading to embryo death with an EC_50_ value of 0.12 μM at 48 h after treatment; mortality at 96 h was 100% even at the lowest concentration of 0.625 μM. All the alkenoic acid derivatives (**1**–**6**) exhibited much lower toxicity on zebrafish embryos. At 48 h, most of the zebrafish embryos were alive when treated with compounds **1**–**6** even at the highest concentration of 10 μM. At 96 h, *bis*-alkenoic acid ester-type compounds (**2**–**4**) and the β-lactone-type compound (**6**) showed anti-proliferative effects on zebrafish embryos at 10 μM.

## 3. Discussion

In this study, four new compounds, including a new alkenoic acid together with three new *bis*-alkenoic acid esters were isolated from the ethyl acetate extract of marine-derived fungus *F. solani* H915. Chemically, the relative configuration of these compounds was confirmed by their NOESY spectra; the absolute configuration was revealed by product elucidation via alkaline hydrolysis experiments. All the compounds were evaluated for antifungal activity. The new compound **4**, containing a β-lactone ring, exhibited moderate inhibitory activities with MIC of 50 μg/disc for *P. theae*. Hymeglusin (**6**) and equisetin (**7**) exhibited potent inhibitory activities against both fungi with an MIC of 25 μg/disc. As two new isolated tea pathogenic fungi, typical antifungal drugs, such as fluconazole and fluorocytosine did not show inhibitory activity on them, even at a concentration of 50 μg/disc. The β-lactone ring containing alkenoic acid derivatives possibly prevents fungal diseases in tea plants.

However, the toxicity of the antifungal regents is important. Although showed potent activity, equisetin exhibited strong anti-proliferative effects on zebrafish embryos and larvae, indicating high toxicity. Alkenoic acid derivatives showed much lower toxicity to zebrafish. Thus, hymeglusin (**6**), an alkenoic acid derivative with a β-lactone ring from *F. solani* H915, is a low-toxicity, potent antifungal agent against tea pathogenic fungi.

## 4. Materials and Methods

### 4.1. General Experimental Procedures

An electrospray ionization source (ESI)-equipped Q-Exactive Mass spectrometer (Thermo Fisher Scientific Corporation, Waltham, MA, USA) was used to analyze the HR-ESI-MS data. A Shimadzu UV-260 spectrometer (Shimadzu Corporation, Tokyo, Japan) and a Perkin-Elmer 683 infrared spectrometer (PerkinElmer, Inc., Waltham, MA, USA) were used to obtain the UV and IR spectra, respectively. A JASCO P-200 polarimeter (JASCO Corporation, Tokyo, Japan) with a 5 cm cell was applied to measure the optical rotation value. The NMR spectra with TMS as the internal standard were taken on a Brucker Avance III 600 FT NMR spectrometer (Bruker Corporation, Billerica, MA, USA).

### 4.2. Fungal Strain and Fermentation

The strain *Fusarium* sp. H915 was isolated from mangrove sediments at the Zhangjiangkou Mangrove National Nature Reserve, Fujian, China, suing the tablet pour method. The internal transcribed spaces (ITS) region was amplified and sequenced using the general primers ITS1 and ITS4. The ITS region of the fungi is a 576 bp DNA sequence (GenBank Accession Number: KY978583) that showed 99% identity to *F. solani*. The strain was deposited at the China Center for Type Culture Collection (CCTCC) as accession number M2017150 and Marine Culture Collection of China (MCCC) as accession number MCCC 3A00957. The fungus grew well on the rice medium in artificial sea water. Carbohydrate fermentation was conducted by subculturing the fungus in rice medium in artificial sea water and incubated at 28 °C for 30 days at a standing position.

### 4.3. Extraction and Isolation

The rice medium (10 kg) of *F. solani* H915 was extracted with ethyl acetate (20 L) trice and concentrated under reduced pressure at 40 °C to yield 16.4 g of the residue.

The EtOAc extract (15 g) was fractionated over a column packed with silica gel (300 g, Yantai Chemical Industry Research Institute, Yantai, China), eluted with petroleum ether-ethyl acetate (*v*/*v*) (20:1; 10:1; 5:1; 2:1; 1:1; each 1.0 L) and chloroform-methyl alcohol (*v*/*v*) (50:1; 20:1; 10:1; 5;1; 2:1; 0:1; each 1.0 L), to afford 10 fractions (Fr. 1–10). Further separation was conducted on the fractions with antifungal activity (Fr. 6 and 8) and the high-yielded fraction (Fr. 9). Fr. 6 (4.6 g) was separated over a Cosmosil reversed-phase C18 (100 g, 75 μm, Nakalai Tesque Co. Ltd., Kyoto, Japan) column and eluted with CH_3_OH/H_2_O (10–100%, each 0.5 L) to provide nine subfractions (Fr. 6-1–Fr. 6-9). Fr. 6-7 (1.3 g) was purified by a preparative Cosmosil octadecylsilane (ODS) column (250 mm × 20.0 mm inner diameter (i.d.), 5 μm, Cosmosil, Nakalai Tesque Co. Ltd., Kyoto, Japan), isocratically eluted with acetonitrile-H_2_O (42:58, *v*/*v*) to obtain compound **6** (300 mg, 1.8% yield). Fr. 6-9 (1.3 g) was separated by the preparative ODS column and eluted with acetonitrile-H_2_O (65:35, *v*/*v*) to obtain compound **7** (57 mg, 0.38% yield). Fr. 8 (1.5 g) was also fractionated by an ODS column and eluted with CH_3_OH/H_2_O in gradient mode and 10 subfractions were obtained (Fr. 8-1–Fr. 8-10). Fr. 8-9 (270 mg) was purified by preparative HPLC column and eluted with acetonitrile-H_2_O (65:35, *v*/*v*) to obtain compound **4** (30 mg, 0.18% yield). Fr. 9 (3.8 g) was also separated over an ODS open-column and eluted with CH_3_OH/H_2_O to yield nine subfractions (Fr. 9-1–Fr. 9-9). Fr. 9-7 (325 mg) was then isolated by preparative ODS column and eluted with acetonitrile-H_2_O (32:68, *v*/*v*) to yield compound **1** (19 mg, 0.11% yield) and compound **5** (14 mg, 0.085% yield). Preparative HPLC purification of Fr. 9-9 (290 mg), eluted with acetonitrile-H_2_O (67:33, *v*/*v*), led to the isolation of compound **2** (10 mg, 0.061% yield) and compound **3** (62 mg, 0.38% yield).

Fusaridioic acid A (**1**): white amorphous powder; [α]${}_{D}^{25}$ value was −6° (*c* = 0.1, CH_3_OH); IR (KBr) (*ν*_max_): 2925, 2355, 1683, 1593, 1253, 1189 and 1079 cm^−1^; UV (MeOH) λ_max_ (log *ε*): 201 (3.70), 230 (3.43) and 270 (3.90) nm. ^1^H NMR (600 MHz, DMSO-*d_6_*) and ^13^C NMR (150 MHz, DMSO-*d_6_*) spectral data were listed in Table 1 and Table 2; HR-ESI-MS: *m*/*z* 341.1968 [M − H^+^]^−^ (calcd. for 341.1964 C_18_H_29_O_6_).

Fusariumester A_1_ (**2**): colorless viscous oil; [α]${}_{D}^{25}$ value was +2° (*c* = 0.1, CH_3_OH); IR (KBr) (*ν*_max_): 3400, 2930, 2864, 2354, 1695, 1632, 1379, 1250 and 1174 cm^−1^; UV (MeOH) λ_max_ (log *ε*): 203 (3.69), 231 (3.47) and 265 (3.74) nm. ^1^H NMR (600 MHz, DMSO-*d_6_*) and ^13^C NMR (150 MHz, DMSO-*d_6_*) spectral data were listed in Table 1 and Table 2; HR-ESI-MS: *m*/*z* 665.3883 [M − H^+^]^−^ (calcd. for 665.3901 C_36_H_57_O_11_).

Fusariumester A_2_ (**3**): colorless viscous oil; [α]${}_{D}^{25}$ value was close to 0° (*c* = 0.1, CH_3_OH); IR (KBr) (*ν*_max_): 2928, 2857, 2361, 2341, 1698, 1603, 1247 and 1175 cm^−1^; UV (MeOH) λ_max_ (log *ε*): 201 (4.17), 233 (3.80) and 270 (4.19) nm. ^1^H NMR (600 MHz, DMSO-*d_6_*) and ^13^C NMR (150 MHz, DMSO-*d_6_*) spectral data were listed in Table 1 and Table 2; HR-ESI-MS: *m*/*z* 665.3882 [M − H^+^]^−^ (calcd. for 665.3901 C_36_H_57_O_11_).

Fusariumester B (**4**): white powder; [α]${}_{D}^{25}$ value was −12° (*c* = 0.1, CH_3_OH); IR (KBr) (ν_max_): 2927, 2661, 2361, 2341, 1823, 1715, 1698, 1616, 1234, 1149 and 1046 cm^−1^; UV (MeOH) λ_max_ (log *ε*): 202 (4.06), 232 (3.80) and 272 (4.23) nm. ^1^H NMR (600 MHz, DMSO-*d_6_*) and ^13^C NMR (150 MHz, DMSO-*d_6_*) spectral data were listed in Table 1 and Table 2. HR-ESI-MS: *m/z* 647.3798 [M − H^+^]^−^ (calcd. for 647.3873 C_36_H_55_O_10_).

### 4.4. Alkaline Hydrolysis of ***2**–**4*** and ***6***

Each compound (1 mg) was dissolved in a mixture of 5% KOH-dioxane (1:1, 4 mL). The solution was stirred at room temperature overnight. The reaction mixture was neutralized with 1% HCl. The neutralized solution was filtered through a 0.22 μM filter membrane to afford the test solution. Each test solution (20 μL) was analyzed over an Cosmosil C18 column (250 mm × 4.6 mm i.d., 5 μm, Nakalai Tesque Co., Ltd., Kyoto Japan) and eluted with water (A) and acetonitrile (B) at a flow rate of 1.0 mL·min^−1^, according to the following gradient program: A from 60% to 40% and B from 40% to 60% during 0–30 min. The detection wavelength was 254 nm. The alkaline hydrolysis products were identified by comparison of their retention times (t_R_) with those of compounds **1** and **5**.

### 4.5. Antifungal Activity

The antifungal activity against tea pathogenic fungi *P. theae* and *C. gloeosporioides* was evaluated according to a previously described method [10,11]. Petri plates were used in the test. A piece of tested fungal strains cylinder agar with diameter of 0.6 cm was placed in the center and sterile blank paper discs (0.5 cm diameter) were placed 2 cm from the growing mycelial colony. Approximately 20 μg of each compound was loaded to each paper disc. DMSO was used as the blank control. The plates were incubated at 28 °C until mycelial growth enveloped the control discs. Then 10 μL of each compound’s DMSO diluted solution ranging from 0.3125 to 10 μg/μL was added onto paper discs. The inoculated plates with impregnated paper discs were incubated at 28 °C for seven days. The lowest concentration of active compound that could inhibit visible mold growth was recorded as the MIC. The experiment was repeated and recorded thrice.

### 4.6. Antiproliferative Effects on Zebrafish Embryo

Compounds **1**–**7** were dissolved in DMSO at a concentration of 10 mM and stored at −20 °C until analysis. Toxic activity of the isolated compounds was analyzed with the anti-proliferative effects on zebrafish *Danio rerio* embryo, according to previously described methods [15]. 3,4-Dichloroaniline was used as a positive control. Fertilized embryos were collected following natural spawning in 12-well plates. Embryos were periodically checked for death and developmental delay. At 6 h post-fertilization (HPF), embryos were arranged in 12-well plates at 20 embryos/well. Pure compounds were then added to the desired concentration DMSO as the vehicle control. DMSO was kept at 0.5% of the total volume. Embryos were grown at 28 °C and examined with a Leica stereomicroscope at 24, 48, 72 and 96 h post treatment. The death rate was recorded every. All animal procedures were conducted in accordance with all appropriate regulatory standards under protocol P18030102 (approval date: 2018-03-01) approved by the Xiamen University Institutional Animal Care and Use Committee.

## Figures and Tables

**Table 1:** ^1^H nuclear magnetic resonance (NMR) (DMSO-*d*_6_, 600 MHz) data of compounds **1**–**6**.

No.	1	2	3	4	5	6 *
2	5.50, br.s	5.60, br.s	5.58, br.s	5.56, br.s	5.50, s	
2′		5.56, br.s	5.57, br.s	5.59, br.s		5.57, br.s
4	5.67, br.s	5.70, br.s	5.71, br.s	5.71, br.s	5.66, s	
4′		5.71, br.s	5.72, br.s	5.76, br.s		5.73, br.s
6a	2.00, dd (13.0, 6.1)	2.02, m	2.05, dd (5.0, 3.3)	2.04, dd (13.1, 6.0)	2.00, dd (13.1, 6.0)	
6b	1.75, dd (13.3, 8.3)	1.82, m	1.82, m	1.80, dd (12.1, 4.4)	1.75, dd (13.1, 8.3)	
6′a		2.06, dd (13.6, 6.2)	2.08, m	2.08, dd (13.3, 6.0)		2.07, dd (13.2, 6.1)
6′b		1.79, m	1.79, m	1.83, dd (13.2, 8.4)		1.83, dd (12.8, 8.3)
7	1.58, m	1.60, m	1.62, m	1.61, m	1.57, br.dd (12.7, 6.4)	
7′		1.60, m	1.62, m	1.64, m		1.64, m
8a	1.18, m	1.24, m	1.24, m	1.25, m	1.19, m	
8b	1.01, m	1.06, m	1.05, m	1.05, m	1.00, m	
8′a		1.24, m	1.24, m	1.28, m		1.28, m
8′b		1.02, m	1.05, m	1.10, m		1.10, m
9a	1.20, m	1.19, m	1.25, m	1.27, m	1.26, m	
9b	1.20, m	1.19, m	1.25, m	1.20, m	1.12, m	
9′a		1.30, m	1.25, m	1.27, m		1.27, m
9′b		1.30, m	1.25, m	1.20, m		1.20, m
10a	1.29, m	1.32, m	1.35, m	1.36, m	1.36, m	
10b	1.20, m	1.20, m	1.19, m	1.20, m	1.15, m	
10′a		1.41, m	1.36, m	1.36, m		1.36, m
10′b		1.26, m	1.24, m			
11a	1.34, m	1.54, m	1.51, m	1.57, m	1.30, m	
11b	1.24, m			1.55, m	1.23, m	
11′a		1.31, m	1.37, m	1.80, m		1.80, m
11′b			1.31, m	1.74, m		1.73, m
12	3.55, m	5.00, m	5.00, td (7.8, 4.7)	5.05, q (6.5)	3.44, td (8.3, 2.9)	
12′		3.50, m	3.62, m	4.53, td (6.6, 4.2)		4.53, td (6.7, 4.2)
13	2.34, dt (8.3, 5.7)	2.57, td (8.3, 4.6)	2.60, td (8.5, 4.9)	2.65, td (8.0, 4.9)	2.29, td (8.5, 4.6)	
13′		2.44, td, (8.8, 4.6)	2.50, m	3.50, br.dd (7.8, 3.9)		3.50, br.dd (7.7, 3.9)
3-CH_3_	2.09, d (1.1)	2.15, br.s	2.15, br.s	2.14, d (0.9)	2.09, d (1.1)	
3′-CH_3_		2.14, br.s	2.16, br.s	2.18, br.s		2.16, d (1.1)
5-CH_3_	1.70, d (1.1)	1.74, br.s	1.75, br.s	1.73, br.s	1.70, d (1.1)	
5′-CH_3_		1.75, br.s	1.76, br.s	1.77, br.s		1.76, d (1.1)
7-CH_3_	0.73, d (6.6)	0.78, d (6.6)	0.78, d (6.6)	0.77, d (6.6)	0.73, d (6.6)	
7′-CH_3_		0.79, d (6.4)	0.79, d (6.6)	0.80, d (6.6)		0.80, d (6.6)
13-CH_2_OH	3.51, dd (10.5, 8.4)	3.60, dd (10.4, 4.5)	3.58, dd (10.5, 9.0)	3.60, dd (10.1, 1.5)	3.66, dd (10.4, 4.5)	
	3.46, dd (10.4, 5.4)	3.53, dd (10.4, 9.0)	3.54, dd (10.5, 4.3)	3.52, dd (10.5, 4.8)	3.55, dd (10.2, 9.1)	
13′-CH_2_OH		3.76, dd (10.4, 4.5)	3.61, dd (10.5, 5.5)	3.72, dd (11.7, 4.2)		3.72, dd (11.7, 4.2)
		3.61, dd (10.4, 9.0)	3.56, dd (10.4, 5.3)	3.63, dd (11.7, 3.3)		3.62, dd (11.7, 3.3)

* In order to compare with the sub-structural unit in **4**, the NMR data are parallel listed with No. 1′ to 13′.

**Table 2:** ^13^C NMR (DMSO-*d*_6_, 150 MHz) data of compounds **1**–**6**.

No.	1	2	3	4	5	No.	2	3	4	6 *
1	168.1	168.3	168.1	170.8	168.1	1′	168.2	168.1	166.1	170.9
2	118.5	119.1	118.7	118.6	118.5	2′	118.8	118.6	117.3	118.6
3	153.2	152.4	153.0	153.1	153.1	3′	152.8	153.1	154.6	153.1
4	129.6	129.7	129.6	129.6	129.6	4′	129.6	129.6	129.5	129.6
5	141.3	140.8	141.2	141.2	141.3	5′	141.1	141.2	142.3	141.3
6	48.9	48.8	48.9	48.6	48.8	6′	48.8	48.8	48.9	48.8
7	30.8	30.7	30.7	30.6	30.8	7′	30.9	30.8	30.8	30.7
8	36.8	36.9	36.9	36.6	36.8	8′	36.4	36.7	36.5	36.6
9	26.8	26.9	26.9	26.3	26.8	9′	26.6	26.7	26.5	26.5
10	25.9	25.0	25.0	25.4	25.8	10′	25.7	26.0	25.2	25.2
11	35.0	32.6	32.6	32.2	35.7	11′	35.8	34.9	33.6	33.6
12	69.3	71.6	71.3	70.9	69.5	12′	69.3	69.3	74.7	74.8
13	55.4	53.3	53.3	52.9	56.0	13′	56.2	55.7	58.8	58.8
14	174.9	174.4	174.1	173.9	175.2	14′	173.3	172.7	168.1	168.1
3-CH_3_	19.5	19.5	19.5	19.5	19.5	3′-CH_3_	19.5	19.5	19.6	19.5
5-CH_3_	18.6	18.5	18.5	18.5	18.6	5′-CH_3_	18.5	18.6	18.6	18.6
7-CH_3_	19.7	19.8	19.7	19.7	19.7	7′-CH_3_	19.7	19.7	19.7	19.7
13-CH_2_OH	60.1	60.6	60.6	60.1	61.3	13′-CH_2_OH	61.5	59.9	56.7	56.7

* In order to compare with the sub-structural unit in **4**, the NMR data are parallel listed with No. 1′ to 13′.

## Supplementary Materials

The following are available online at http://www.mdpi.com/1660-3397/16/12/483/s1, Figure S1–S8: Spectra of compound **1**, Figure S9–S17: Sepctra of compound **2**, Figure S18–S26: Spectra of compound **3**, Figure S27–S34: Spectra of compound **4**, Figure S35: HPLC chromatograms of compounds **1**–**6** and alkaline hydrolysis products of **2**–**4**, **6**.
