# Supplementary material for: New Bis-Alkenoic Acid Derivatives from a Marine-Derived Fungus Fusarium solani H915
Source: Mar Drugs. 2018 Dec 3;16(12):483. doi: 10.3390/md16120483 (PMC6316767; doi:10.3390/md16120483)
Supplement: Supplementary file 1 [file marinedrugs-16-00483-s001.pdf]

# Supplementary Information

| Content | Page |
|---|---|
| Figure S1. HR-ESI-MS spectra of compound **1** | 2 |
| Figure S2. IR spectrum of compound **1** | 3 |
| Figure S3. $^1$H-NMR (600 MHz, DMSO-$d_6$) of compound **1** | 4 |
| Figure S4. $^{13}$C-NMR (150 MHz, DMSO-$d_6$) of compound **1** | 5 |
| Figure S5. DEPT spectrum of compound **1** | 6 |
| Figure S6. HSQC spectrum of compound **1** | 7 |
| Figure S7. $^1$H-$^1$H COSY spectrum of compound **1** | 8 |
| Figure S8. HMBC spectrum of compound **1** | 9 |
| Figure S9. HR-ESI-MS spectra of compound **2** | 10 |
| Figure S10. IR spectrum of compound **2** | 11 |
| Figure S11. $^1$H-NMR (600 MHz, DMSO-$d_6$) of compound **2** | 12 |
| Figure S12. $^{13}$C-NMR (150 MHz, DMSO-$d_6$) of compound **2** | 13 |
| Figure S13. DEPT spectrum of compound **2** | 14 |
| Figure S14. HSQC spectrum of compound **2** | 15 |
| Figure S15. $^1$H-$^1$H COSY spectrum of compound **2** | 16 |
| Figure S16. HMBC spectrum of compound **2** | 17 |
| Figure S17. NOESY spectrum of compound **2** | 18 |
| Figure S18. HR-ESI-MS spectra of compound **3** | 19 |
| Figure S19. IR spectrum of compound **3** | 20 |
| Figure S20. $^1$H-NMR (600 MHz, DMSO-$d_6$) of compound **3** | 21 |
| Figure S21. $^{13}$C-NMR (150 MHz, DMSO-$d_6$) of compound **3** | 22 |
| Figure S22. DEPT spectrum of compound **3** | 23 |
| Figure S23. HSQC spectrum of compound **3** | 24 |
| Figure S24. $^1$H-$^1$H COSY spectrum of compound **3** | 25 |
| Figure S25. HMBC spectrum of compound **3** | 26 |
| Figure S26. NOESY spectrum of compound **3** | 27 |
| Figure S27. HR-ESI-MS spectra of compound **4** | 28 |
| Figure S28. IR spectrum of compound **4** | 29 |
| Figure S29. $^1$H-NMR (600 MHz, DMSO-$d_6$) of compound **4** | 30 |
| Figure S30. $^{13}$C-NMR (150 MHz, DMSO-$d_6$) of compound **4** | 31 |
| Figure S31. DEPT spectrum of compound **4** | 32 |
| Figure S32. HSQC spectrum of compound **4** | 33 |
| Figure S33. $^1$H-$^1$H COSY spectrum of compound **4** | 34 |
| Figure S34. HMBC spectrum of compound **4** | 35 |
| Figure S35. HPLC chromatograms of compounds **1-6** and alkaline hydrolysis products of **2-4**, **6** | 36 |

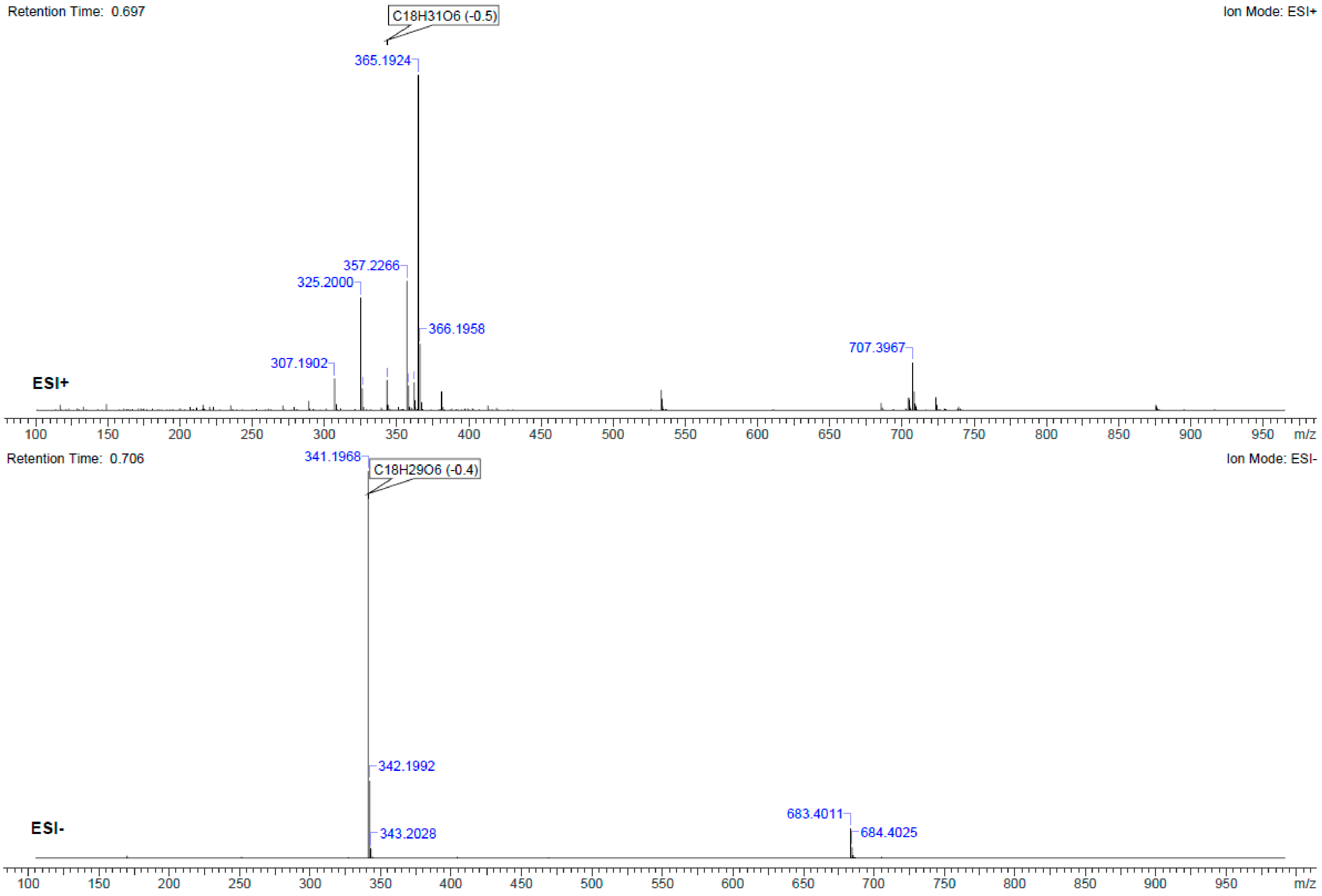


Figure S 1. HR-ESI-MS spectra of compound 1

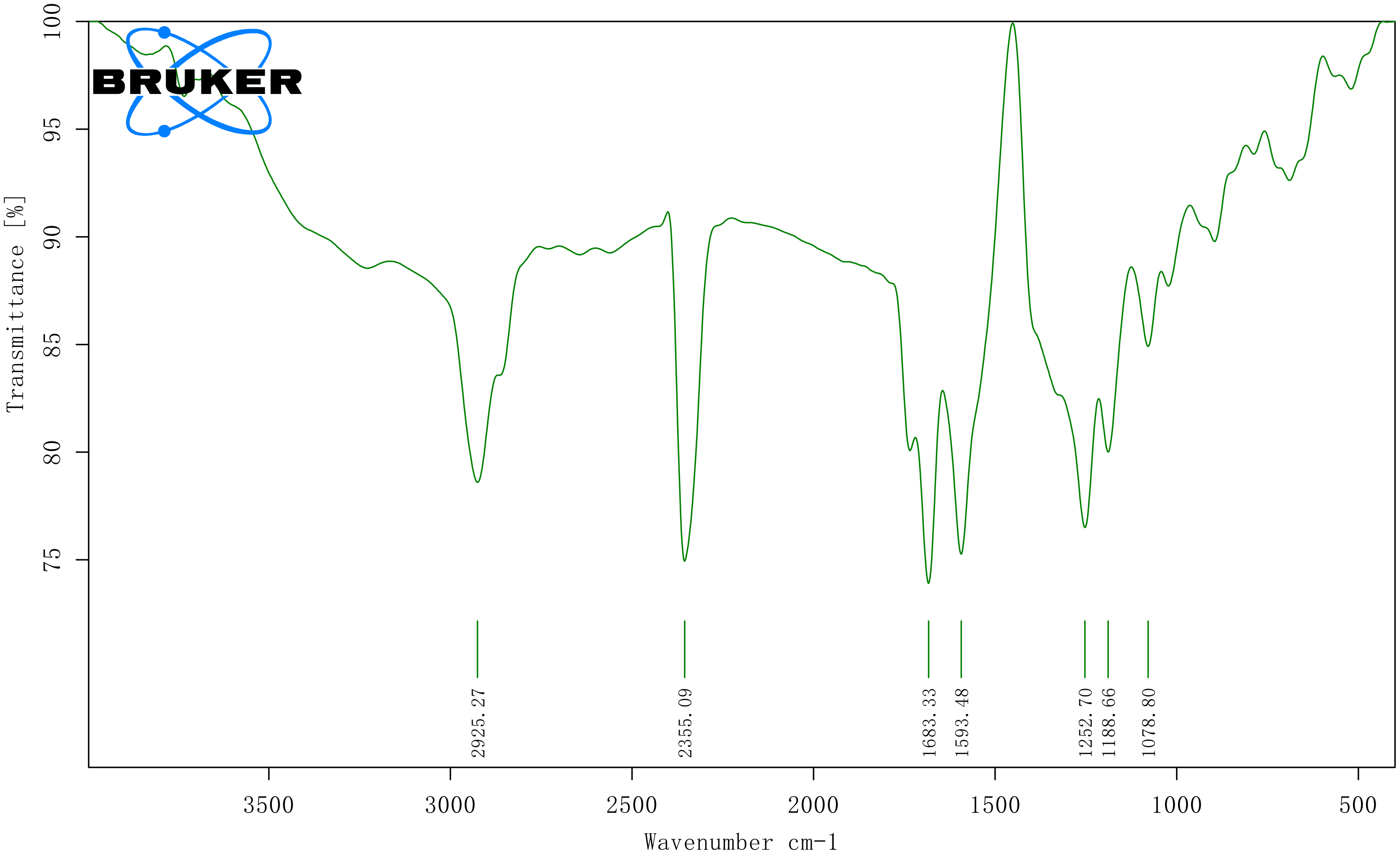


| | | | |
|---|---|---|---|
| D:\My Documents\Bruker\OPUS_7.5.18\DATA\MEAS\GO-3.20 | GO-3 | Instrument type and / or accessory | 2018-8-18 |



Figure S 2. IR spectrum of compound 1

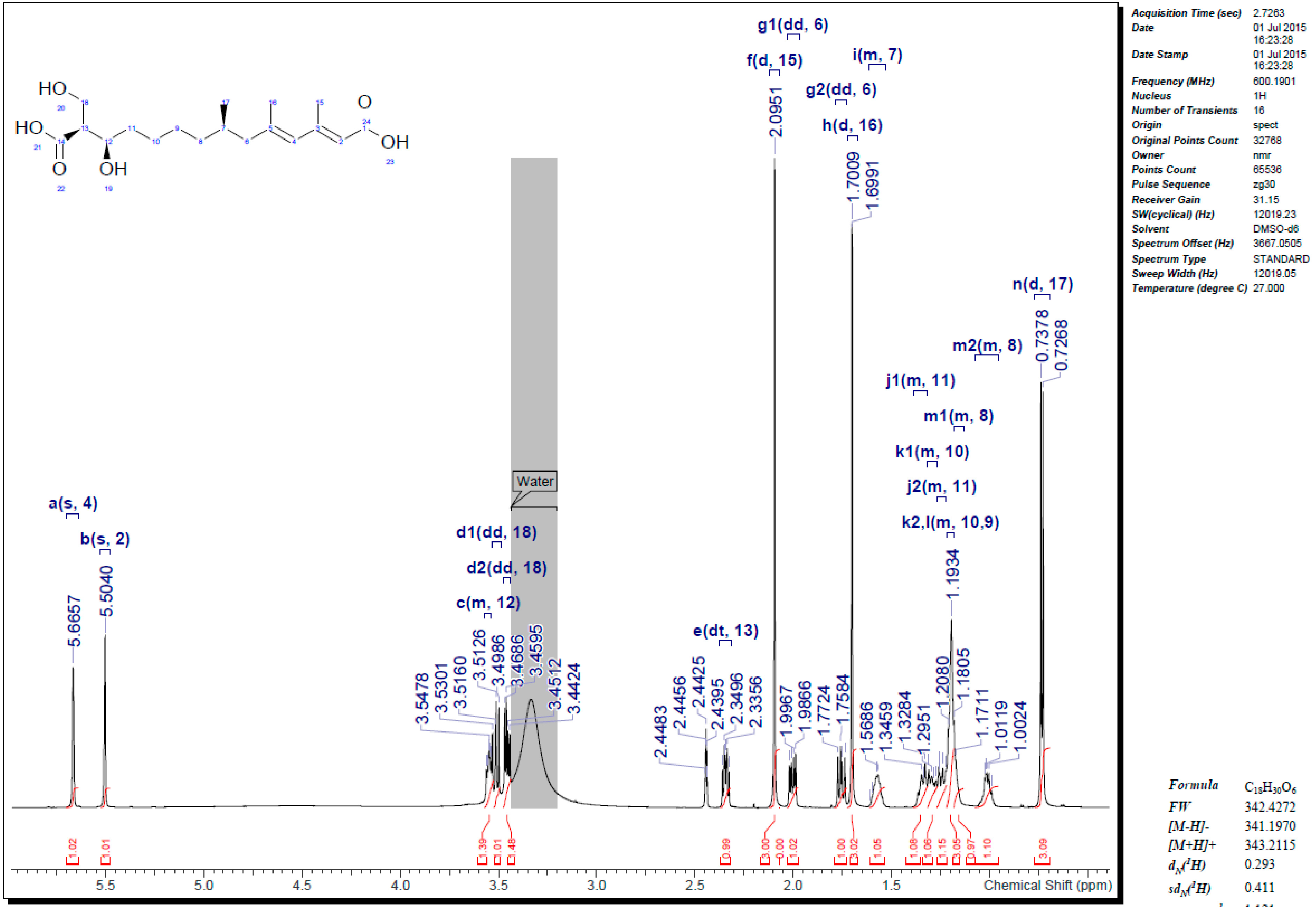

Figure S 3. $^{1}$H-NMR (600 MHz, DMSO-$d_6$) of compound **1**

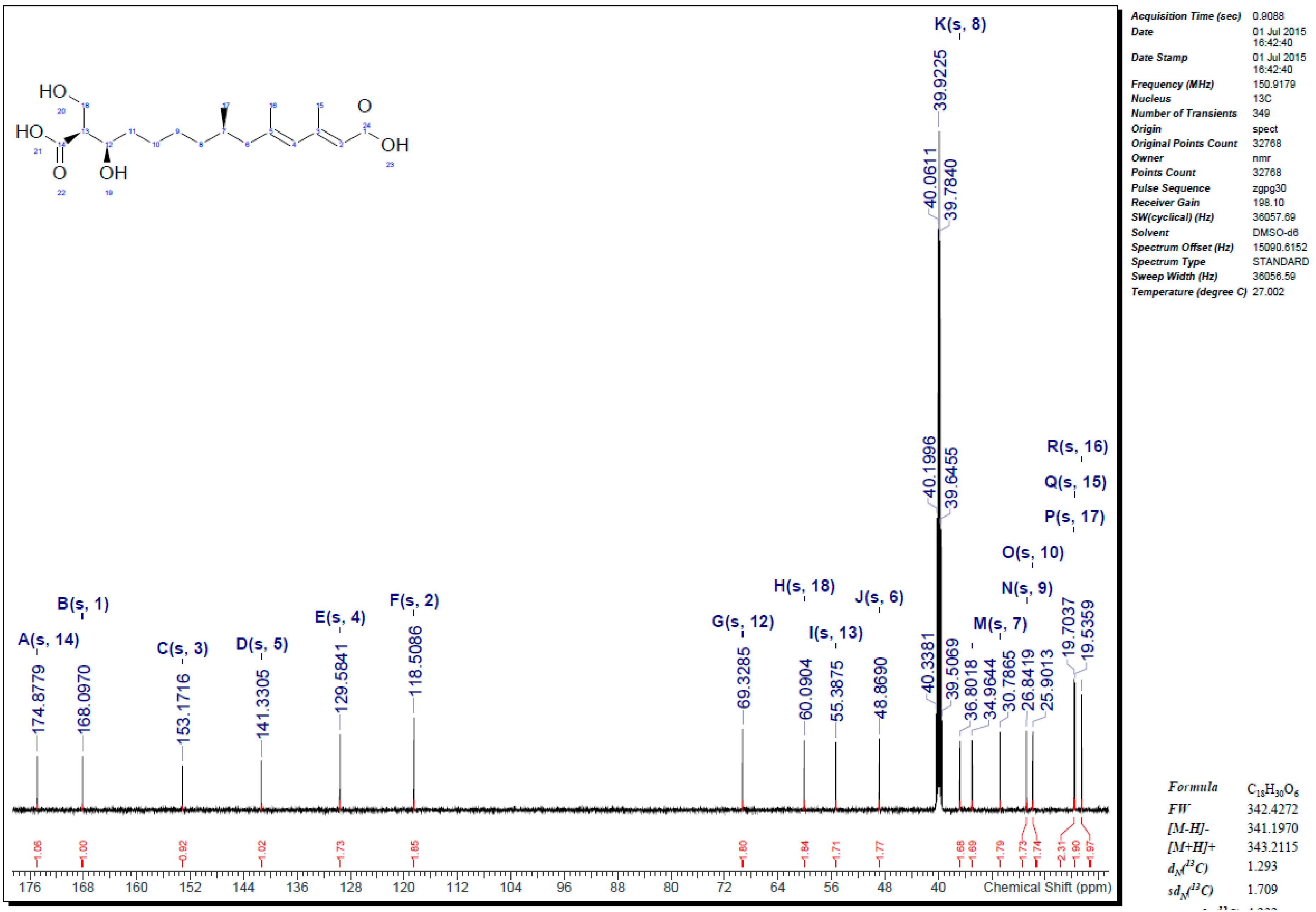


Figure S 4. $^{13}$C-NMR (150 MHz, DMSO-$d_6$) of compound **1**

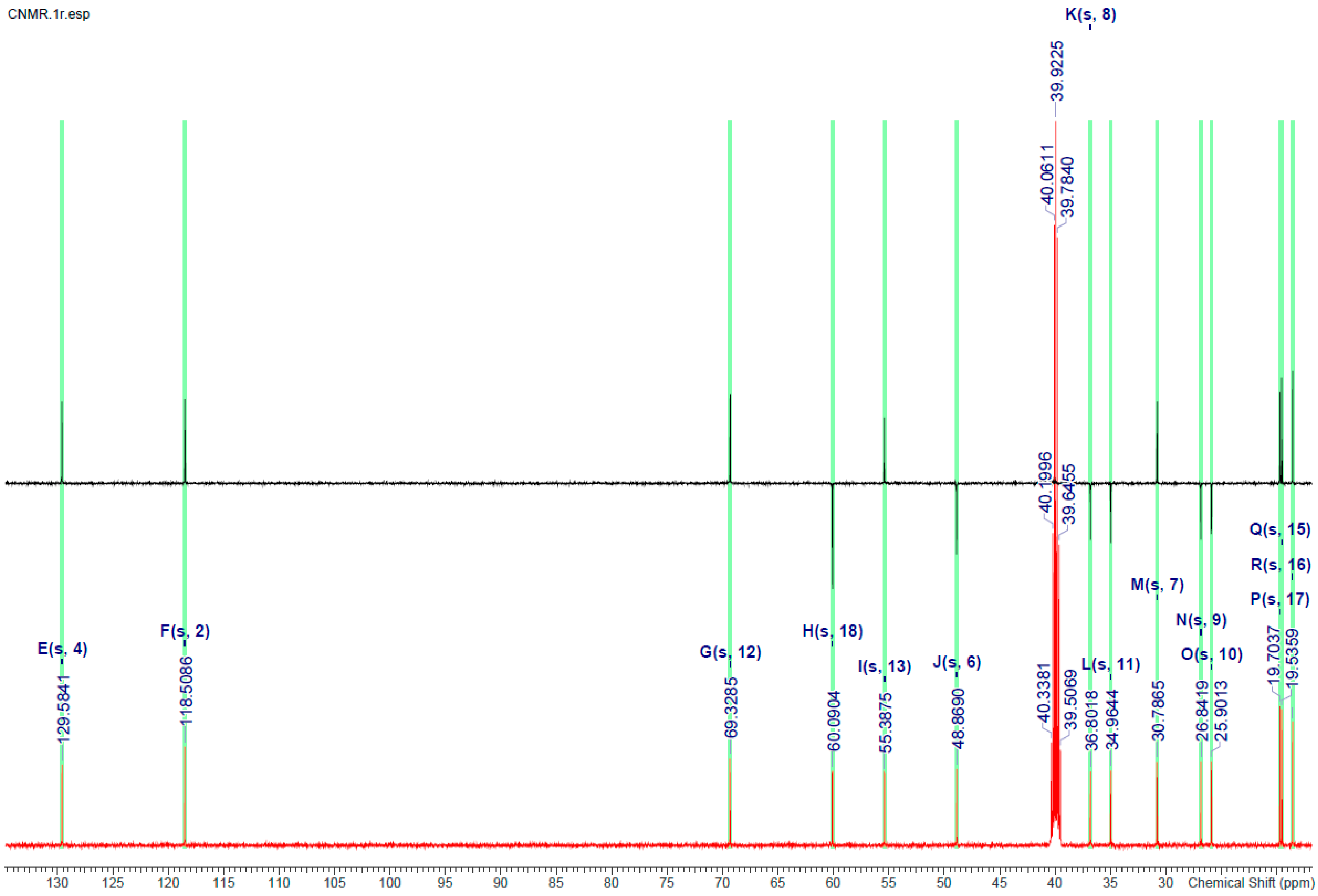


Figure S 5. DEPT spectrum of compound **1**

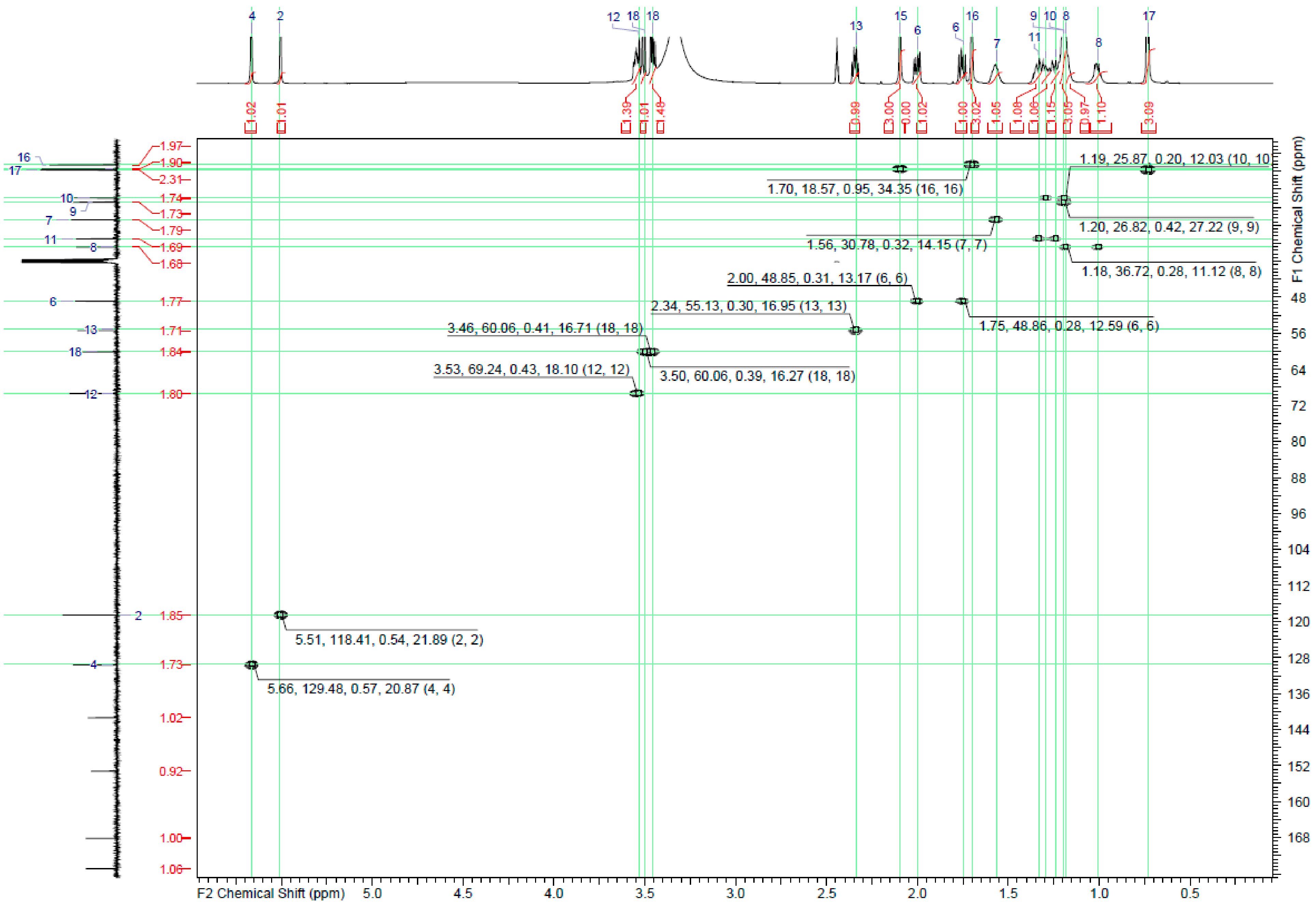


Figure S 6. HSQC spectrum of compound **1**

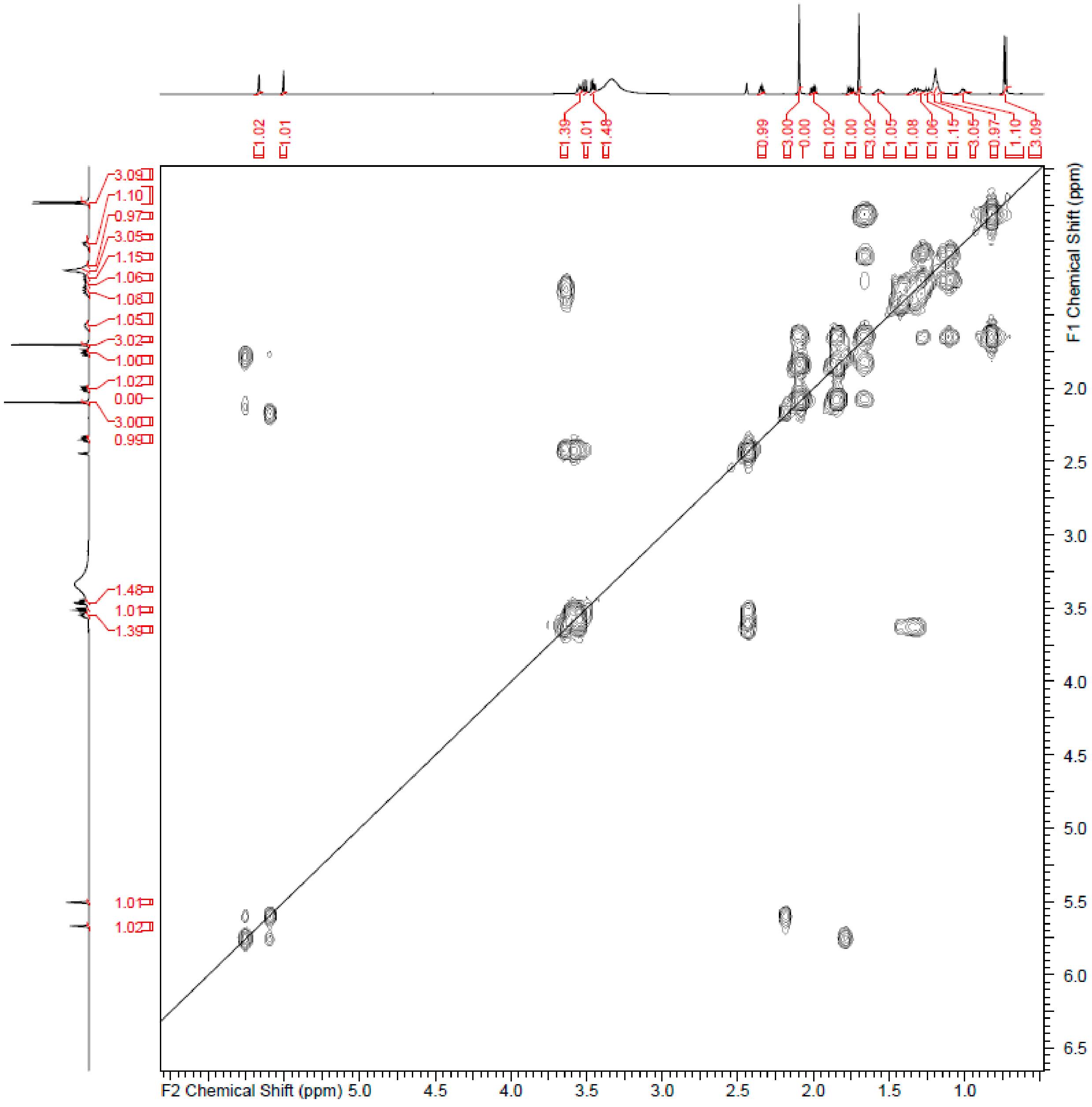


| | |
|---|---|
| *Acquisition Time (sec)* | (0.0852, 0.0214) |
| *Comment* | 5 mm PABBO BB/19F-1H/D Z-GRD Z114607/0133 |
| *Date* | 04 Jan 2017 07:35:50 |
| *File Name* | C:\Users\Administrator\Desktop\yanxia-20170104\H915CM5-1-70%-4\3\pdata\1\2rr |
| *Frequency (MHz)* | (600.1936, 600.1936) |
| *Nucleus* | (1H, 1H) |
| *Number of Transients* | 4 |
| *Origin* | spect |
| *Original Points Count* | (1024, 256) |
| *Owner* | nmr |
| *Points Count* | (2048, 1024) |
| *Pulse Sequence* | cosygpmfqf |
| *Solvent* | DMSO-d6 |
| *Spectrum Type* | COSY |
| *Sweep Width (Hz)* | (12013.36, 11978.70) |
| *Temperature (degree C)* | 23.964 |
| *Title* | |

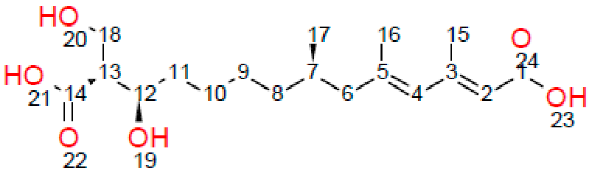


Figure S 7. $^{1}$H-$^{1}$H COSY spectrum of compound **1**

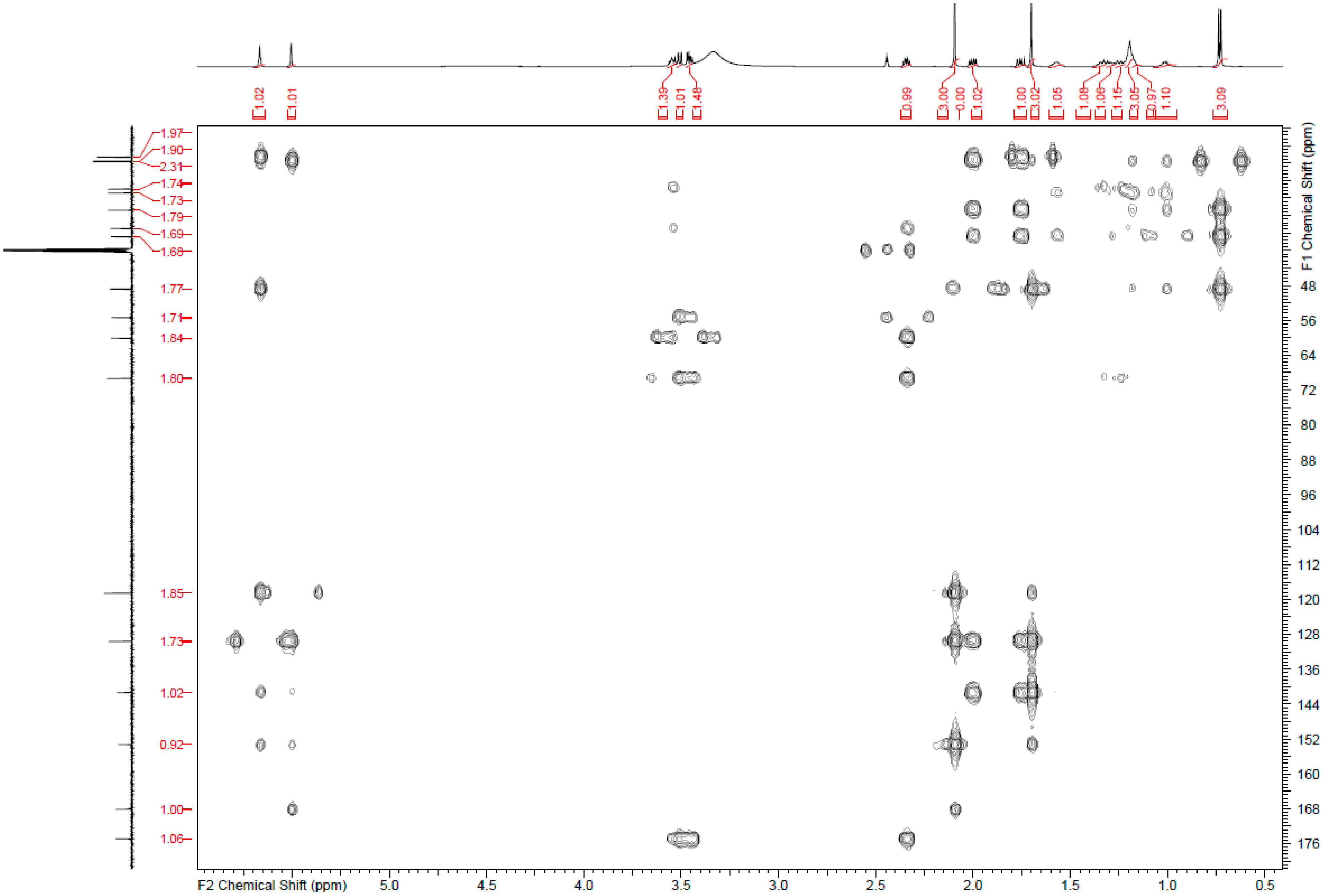


Figure S 8. HMBC spectrum of compound **1**

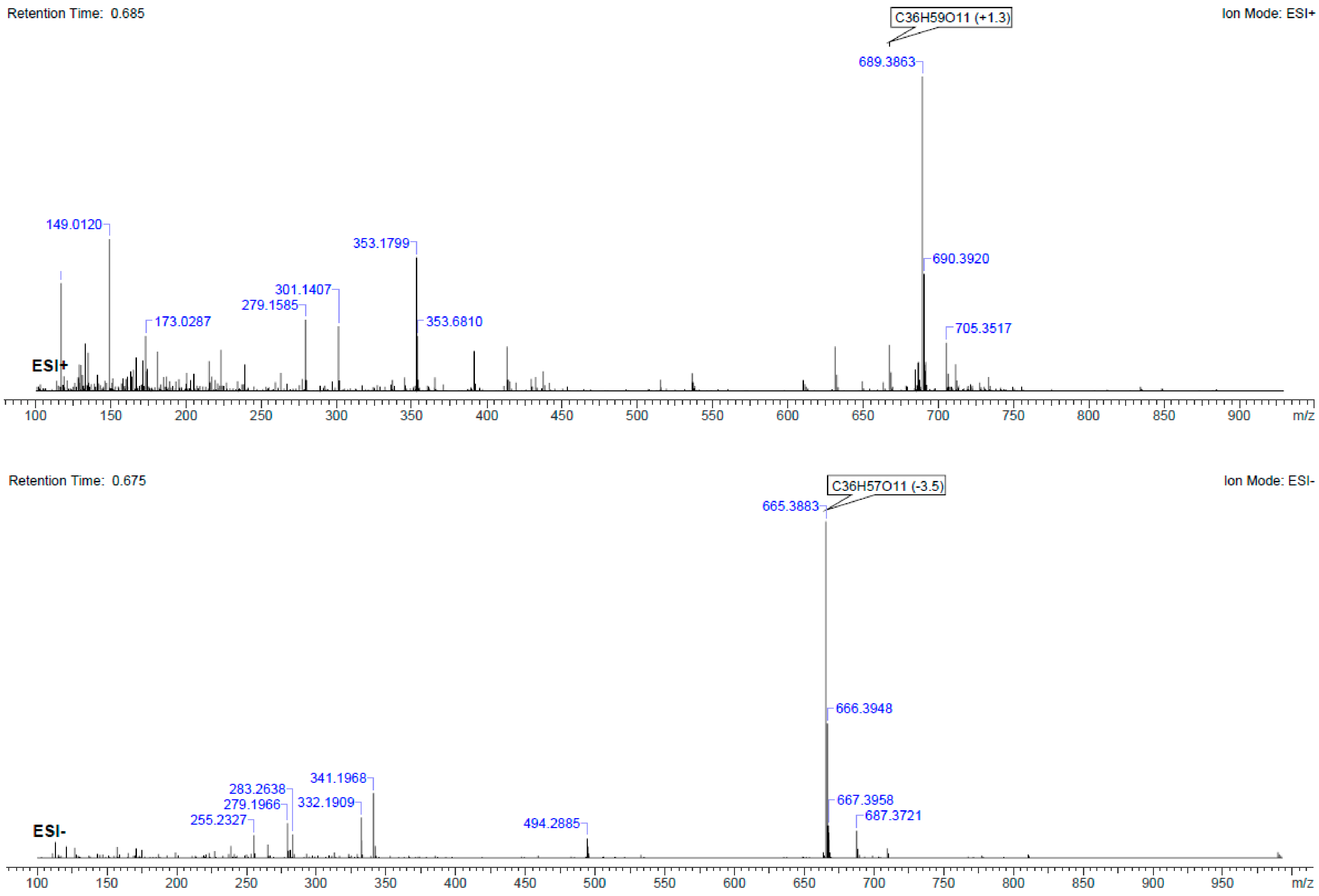


Figure S 9. HR-ESI-MS spectra of compound **2**

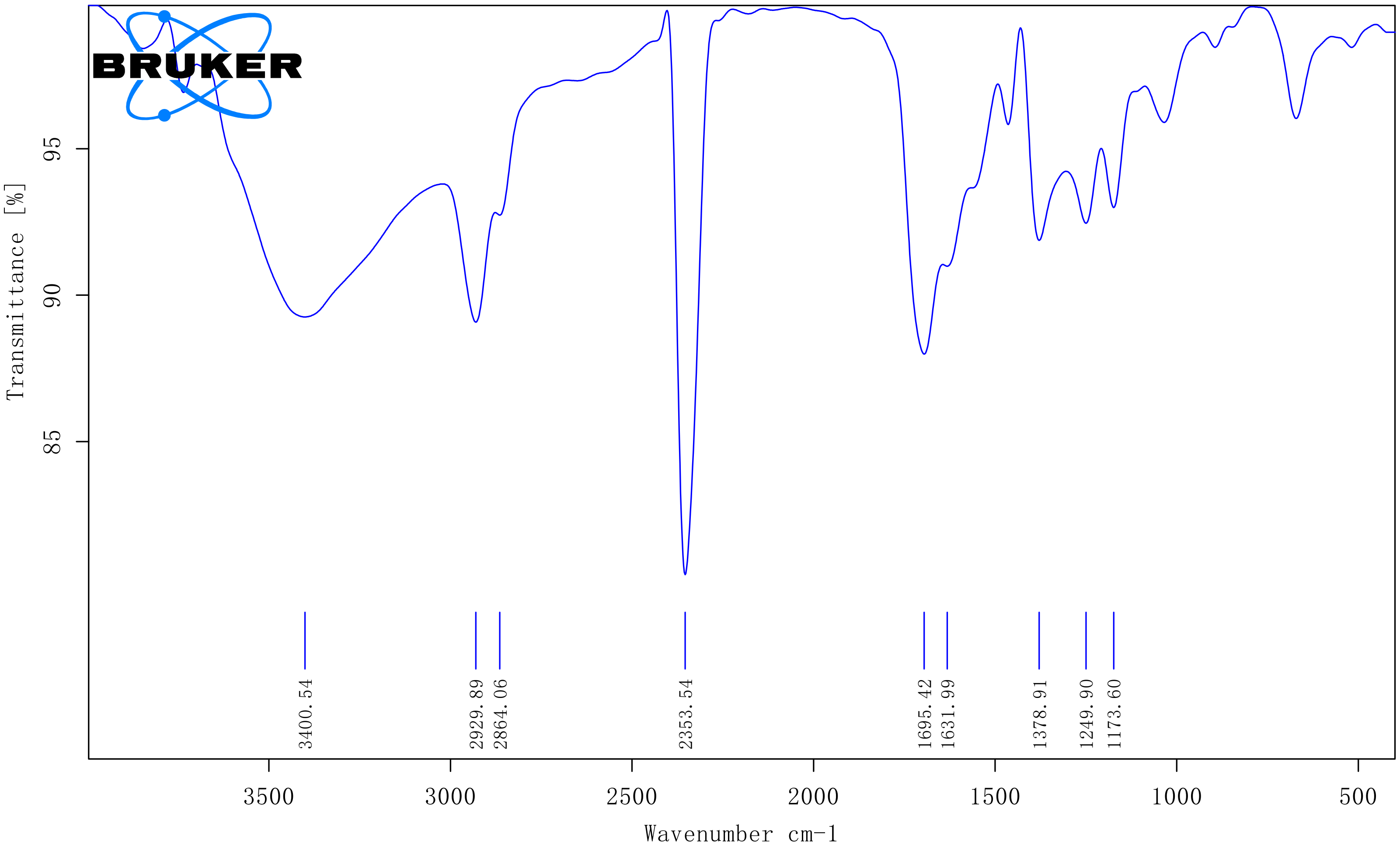


| | | | |
|---|---|---|---|
| D:\My Documents\Bruker\OPUS_7.5.18\DATA\MEAS\GO-3.25 | GO-3 | Instrument type and / or accessory | 2018-8-20 |

**Page 1 of 1**

Figure S 10. IR spectrum of compound **2**

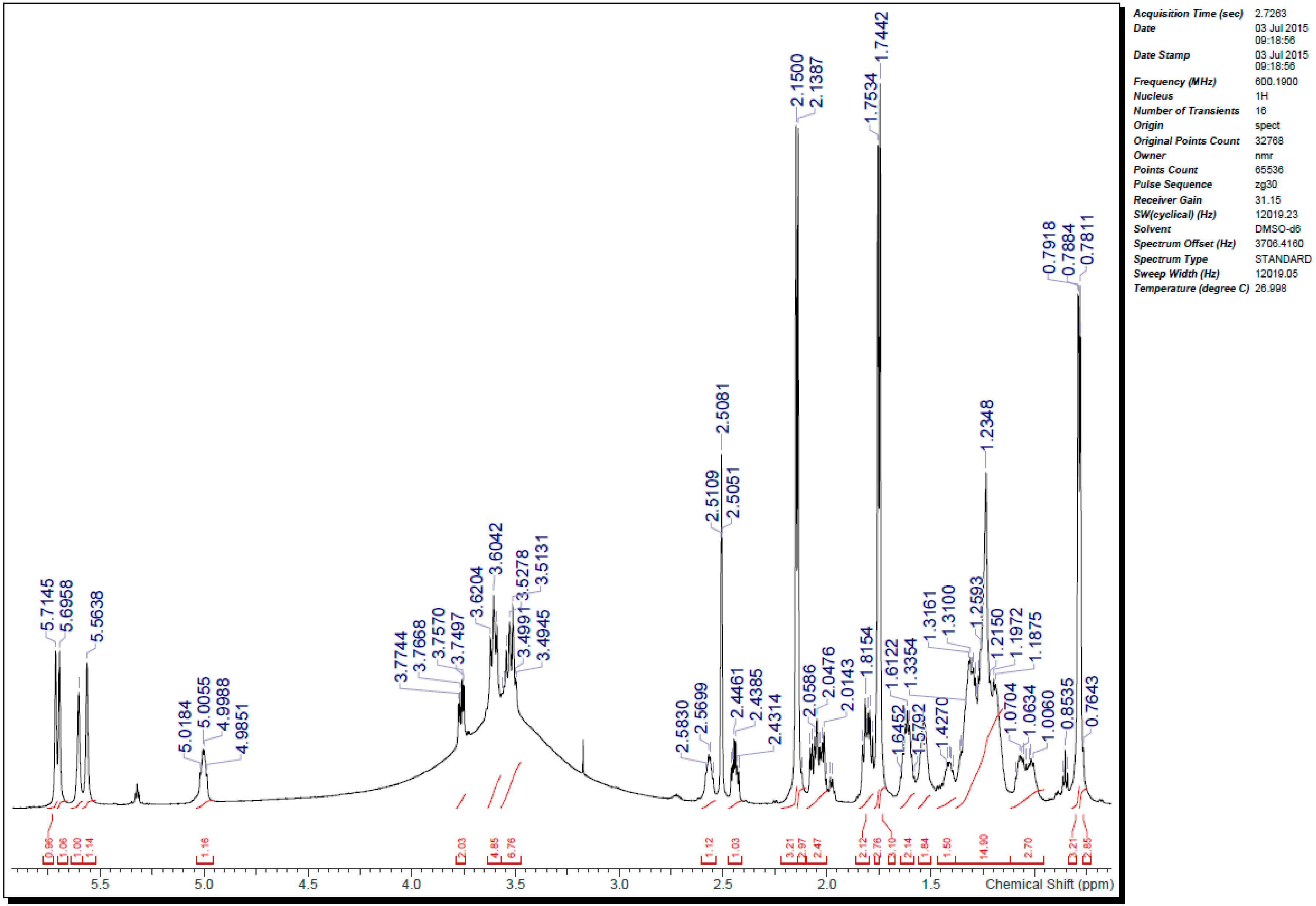


Figure S 11. $^{1}$H-NMR (600 MHz, DMSO-$d_6$) of compound **2**

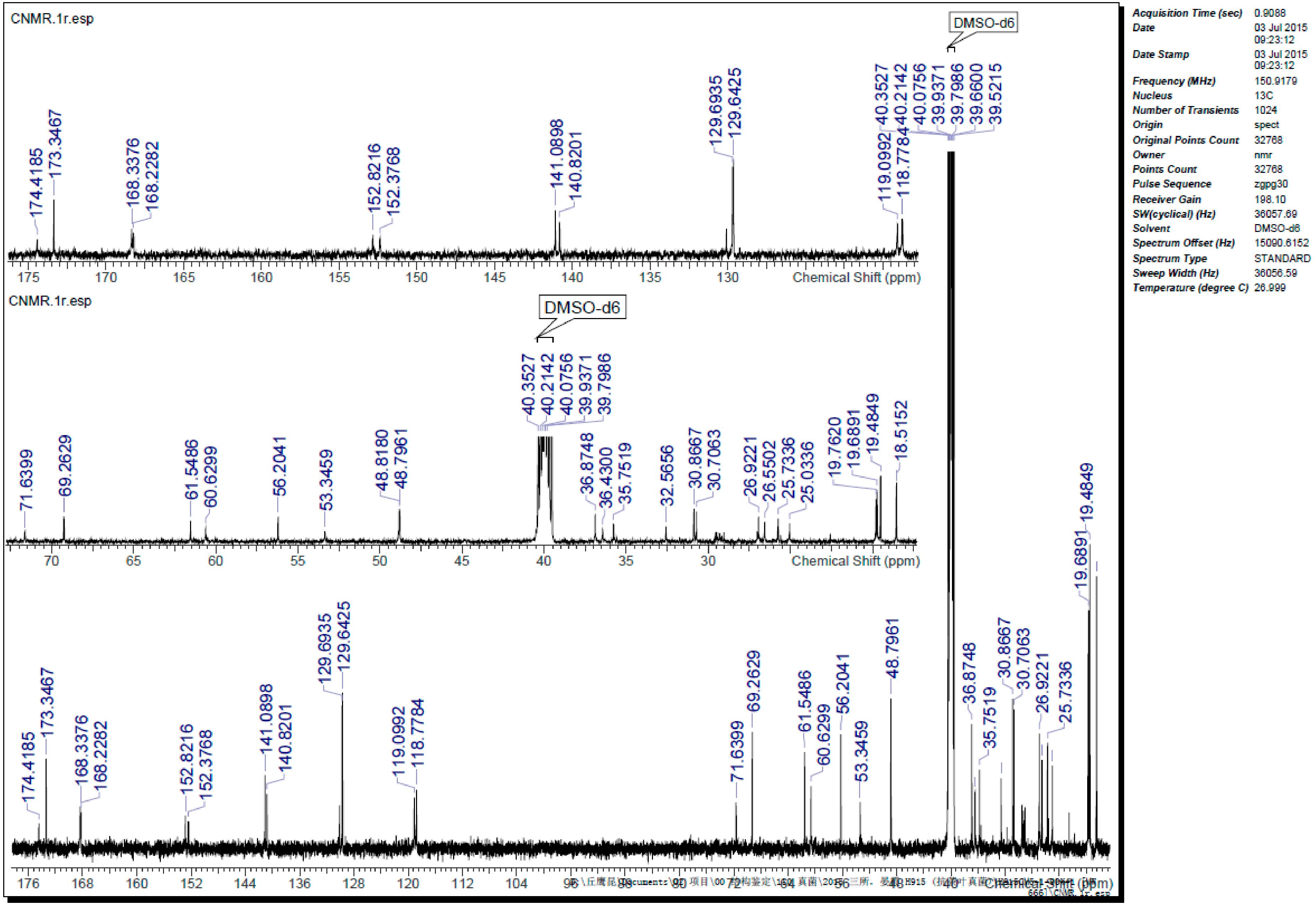


Figure S 12. $^{13}$C-NMR (150 MHz, DMSO-$d_6$) of compound **2**

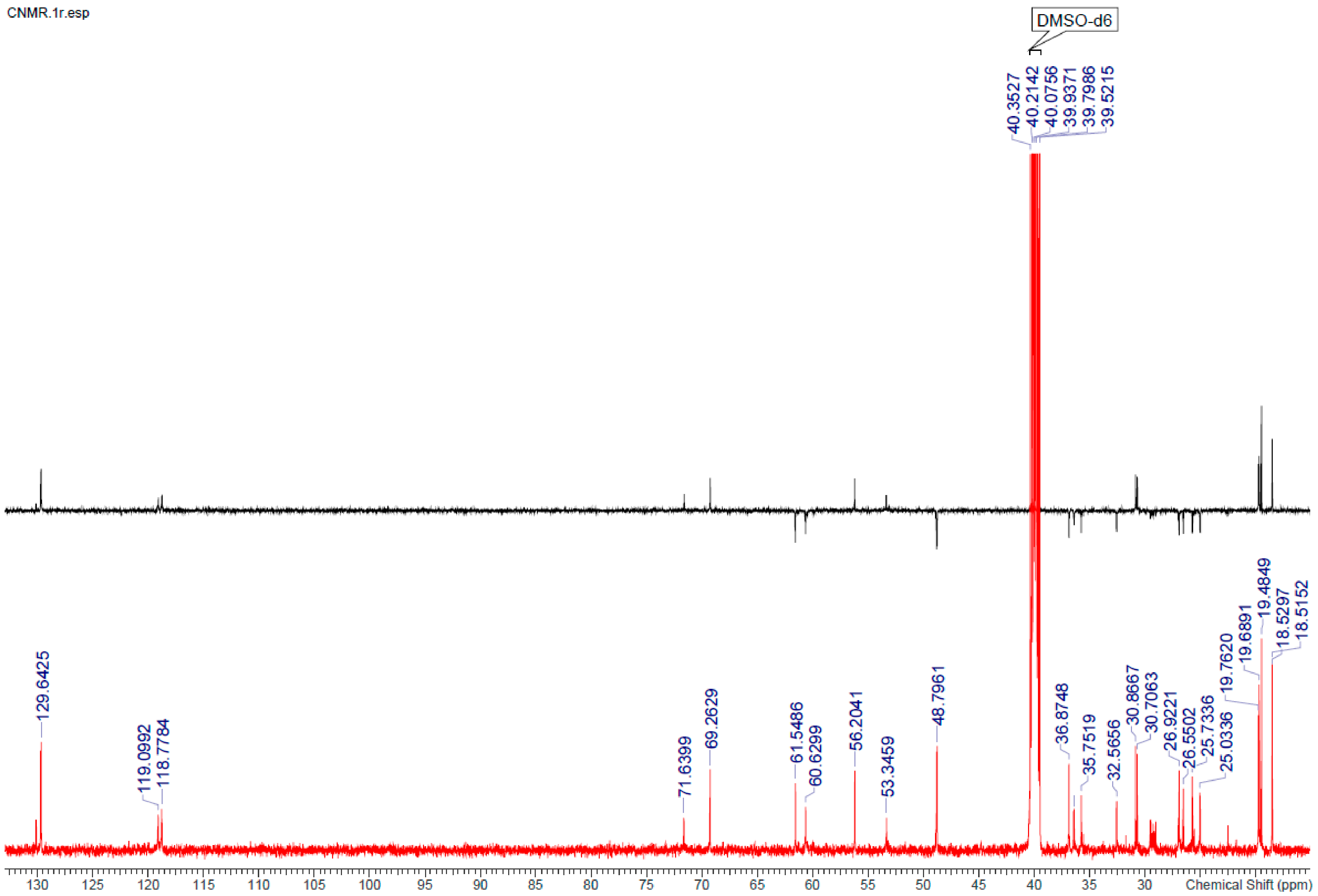


Figure S 13. DEPT spectrum of compound **2**

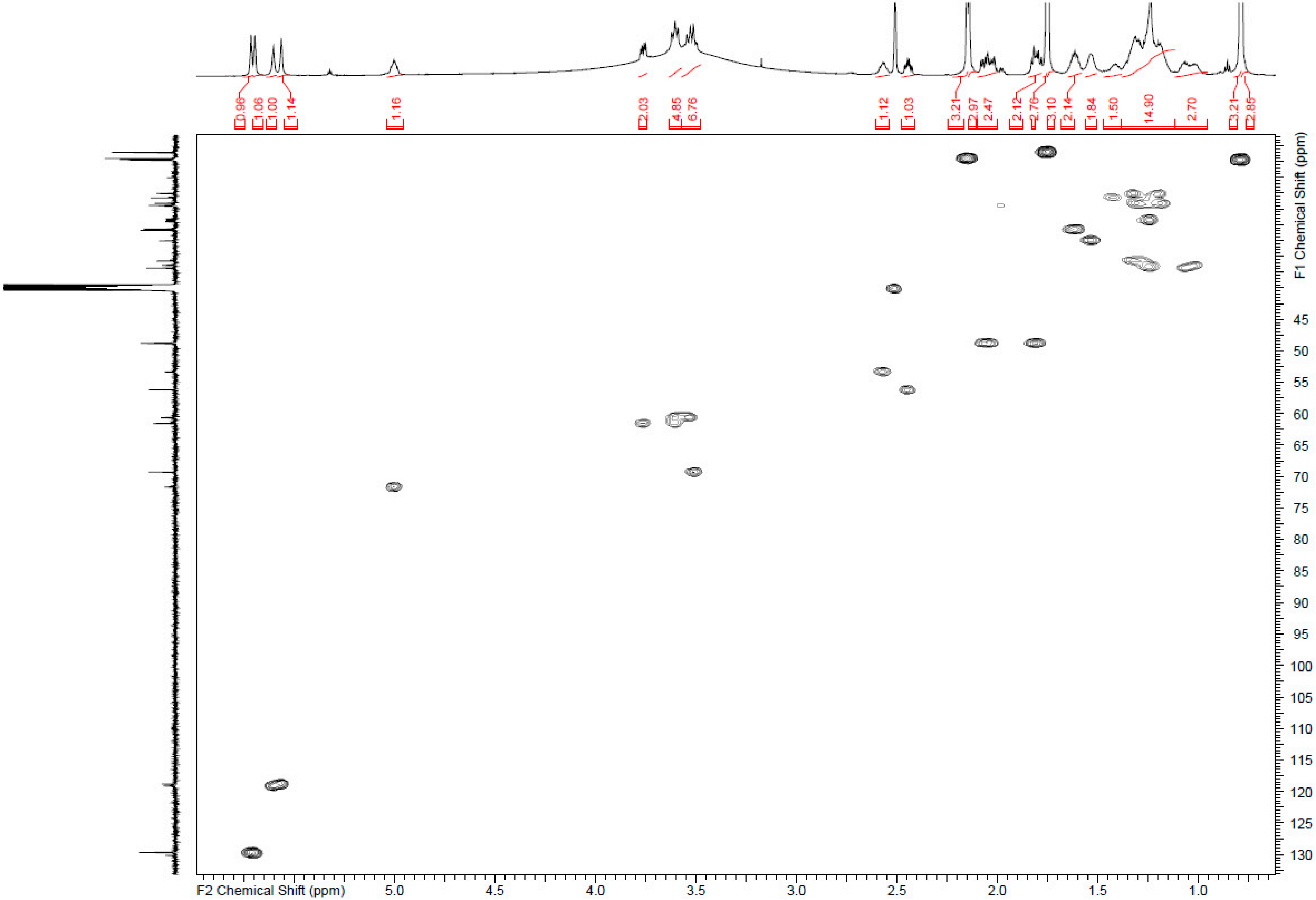


Figure S 14. HSQC spectrum of compound **2**

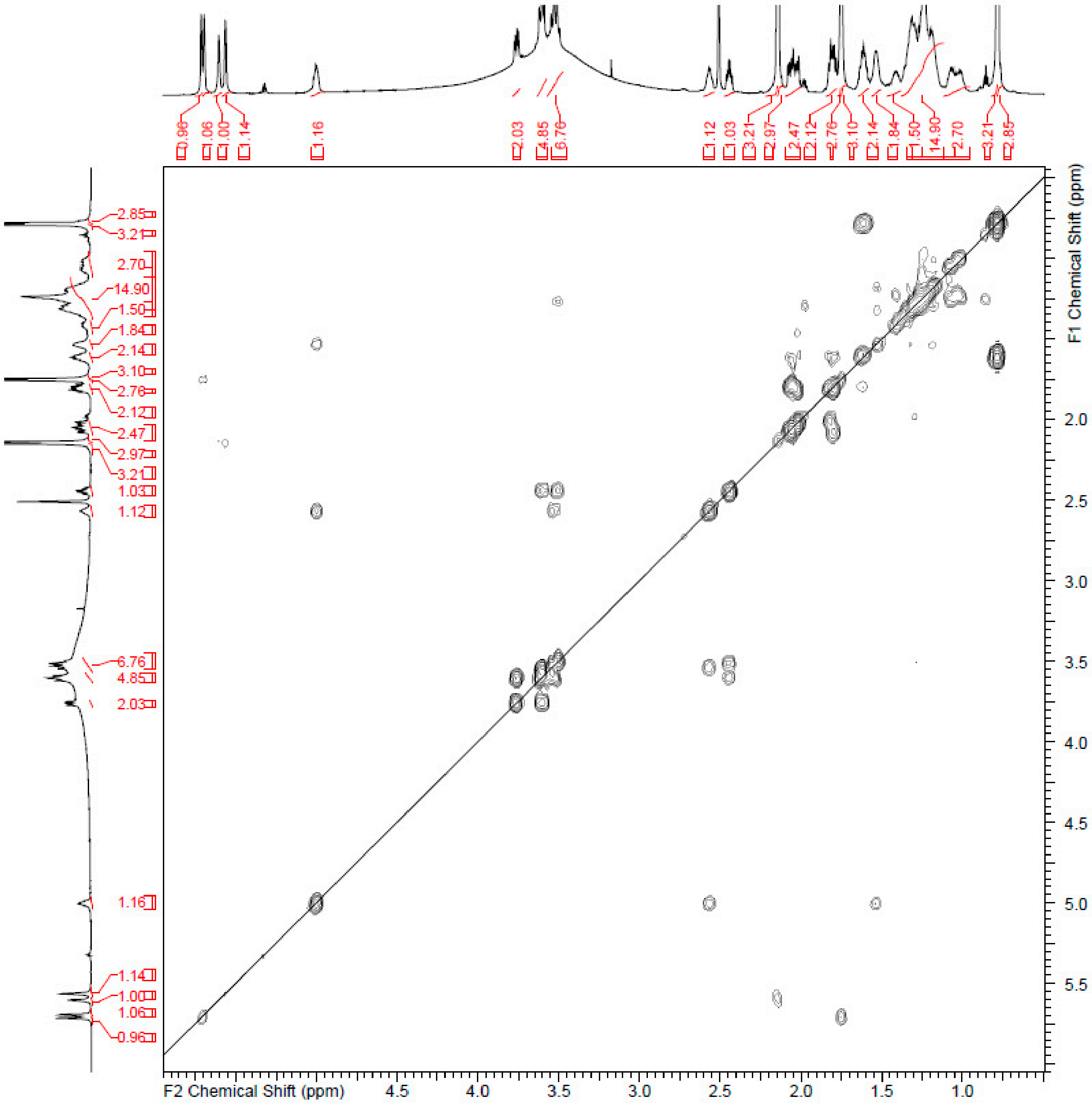

| | |
|---|---|
| *Acquisition Time (sec)* | (0.0852, 0.0214) |
| *Comment* | 5 mm PABBO BB/19F-1H/D Z-GRD Z114607/0133 |
| *Date* | 24 Nov 2016 15:30:38 |
| *File Name* | C:\Users\Administrator\Desktop\yanxia-20161125\H915CM5-1-90%-1\3\pdata\1\2rr |
| *Frequency (MHz)* | (600.1936, 600.1936) |
| *Nucleus* | (1H, 1H) |
| *Number of Transients* | 4 |
| *Origin* | spect |
| *Original Points Count* | (1024, 256) |
| *Owner* | nmr |
| *Points Count* | (2048, 1024) |
| *Pulse Sequence* | cosygpmfqf |
| *Solvent* | DMSO-d6 |
| *Spectrum Type* | COSY |
| *Sweep Width (Hz)* | (12013.36, 11978.70) |
| *Temperature (degree C)* | 23.966 |
| *Title* | |

Figure S 15. $^{1}H$-$^{1}H$ COSY spectrum of compound **2**

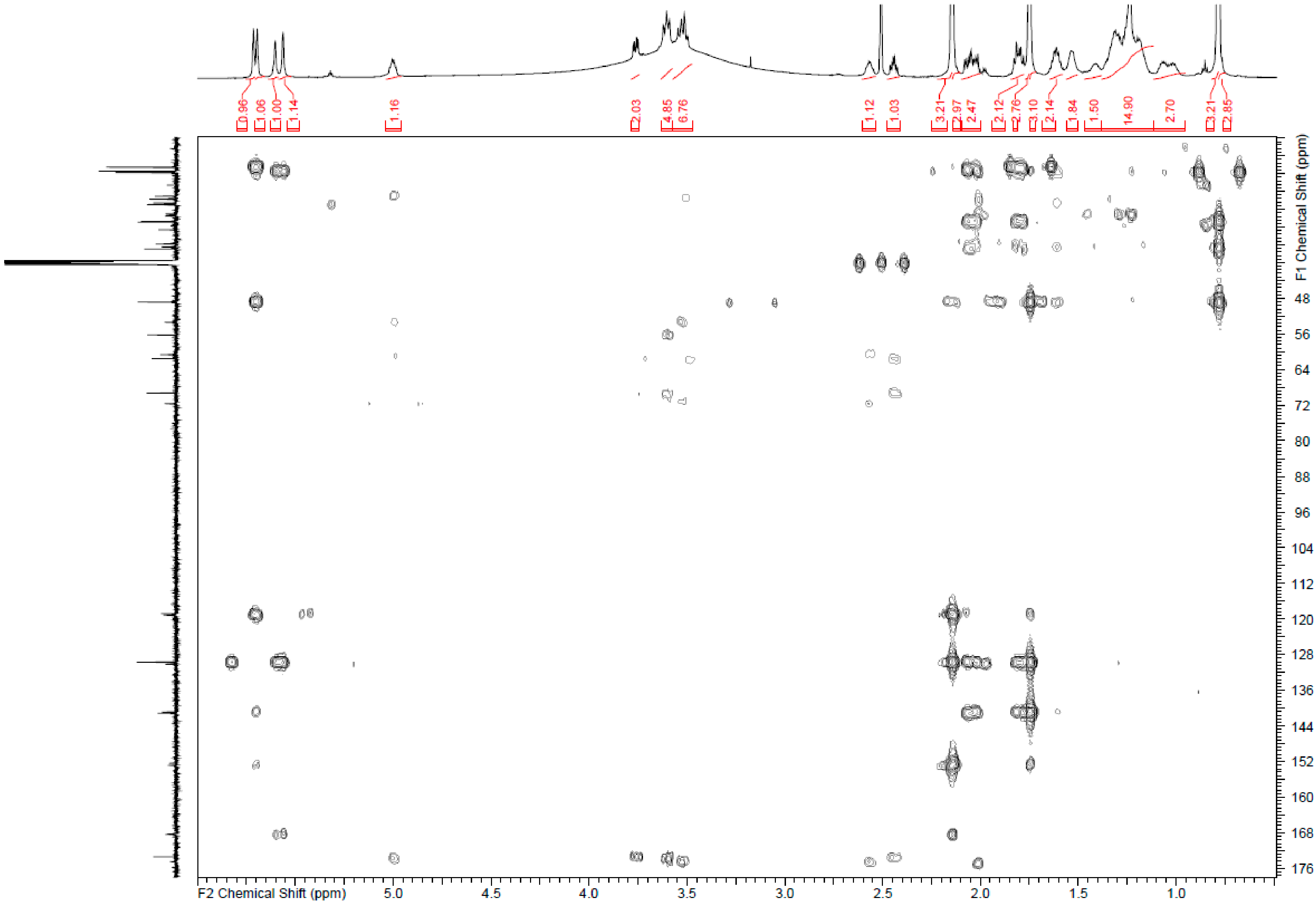


Figure S 16. HMBC spectrum of compound **2**

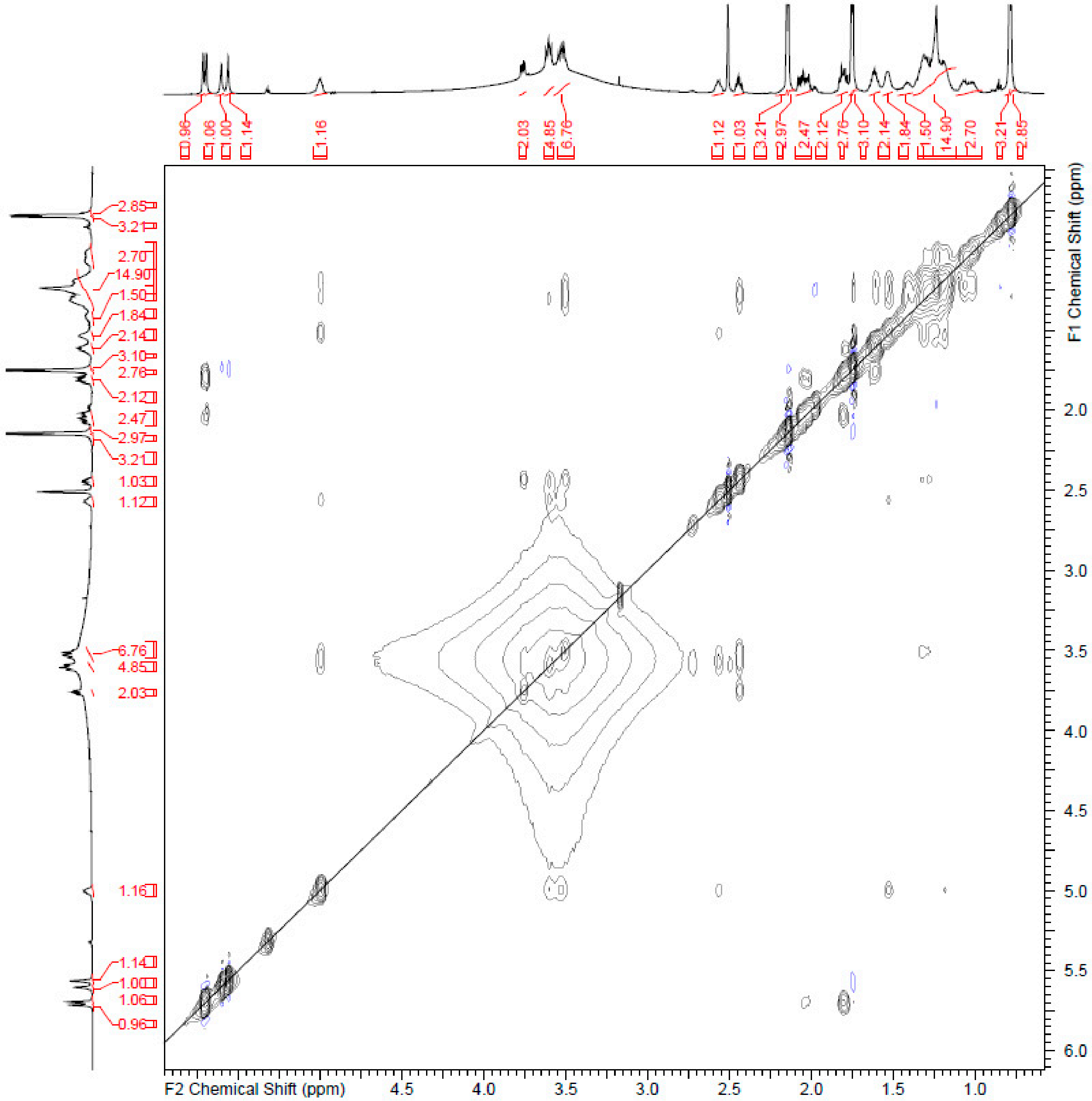

| | |
|---|---|
| *Acquisition Time (sec)* | (0.1454, 0.0364) |
| *Comment* | 5 mm PABBO BB/19F-1H/D Z-GRD Z114607/0133 |
| *Date* | 25 Nov 2016 08:55:30 |
| *File Name* | C:\Users\Administrator\Desktop\yanxia-20161125\H915CM5-1-90%-1\6\pdata\1\2rr |
| *Frequency (MHz)* | (600.1921, 600.1921) |
| *Nucleus* | (1H, 1H) |
| *Number of Transients* | 4 |
| *Origin* | spect |
| *Original Points Count* | (1024, 256) |
| *Owner* | nmr |
| *Points Count* | (1024, 1024) |
| *Pulse Sequence* | noesygpphpp |
| *Solvent* | DMSO-d6 |
| *Spectrum Type* | NOESY |
| *Sweep Width (Hz)* | (7035.38, 7035.38) |
| *Temperature (degree C)* | 24.002 |
| *Title* | |

Figure S 17. NOESY spectrum of compound **2**

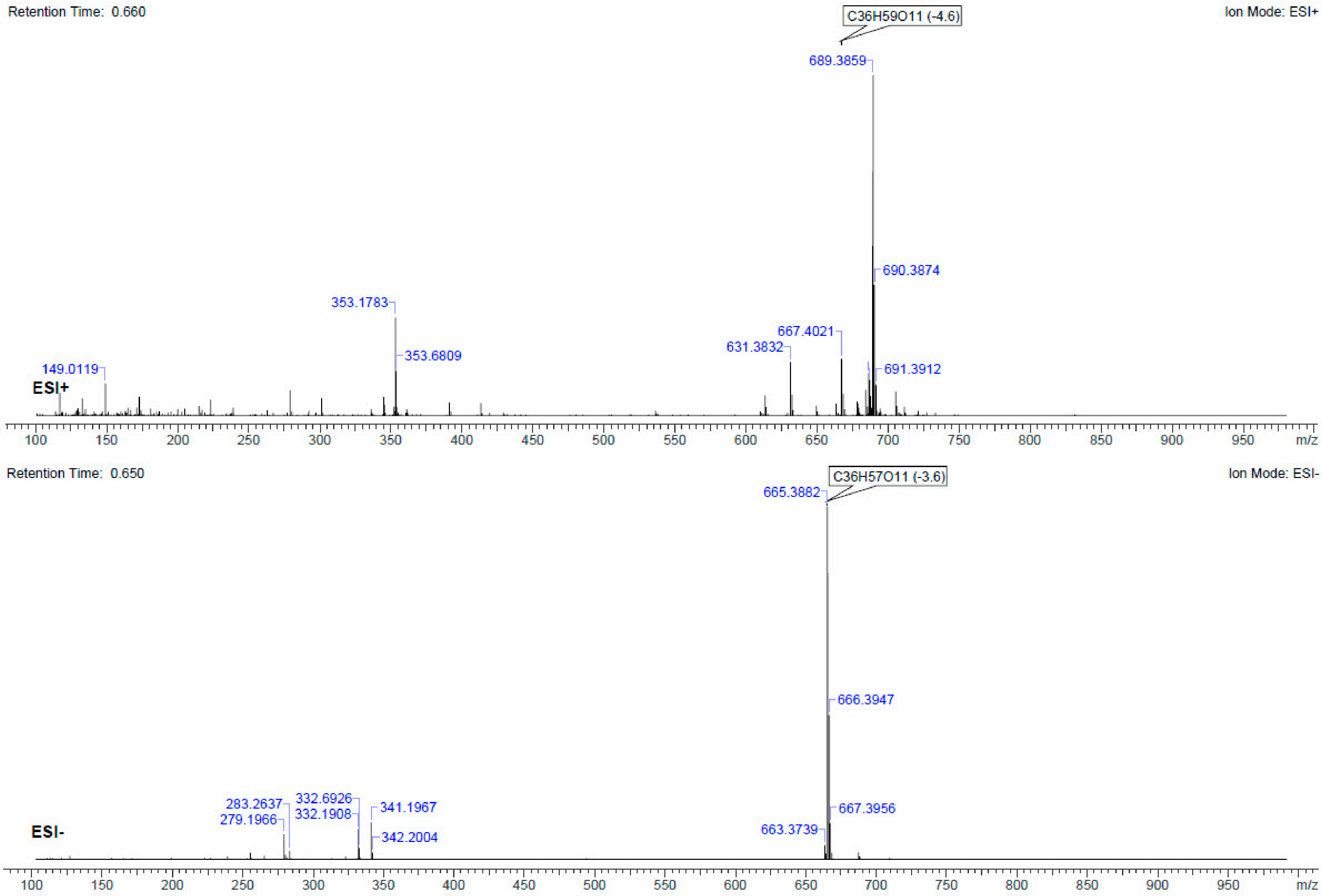


Figure S 18. HR-ESI-MS spectra of compound **3**

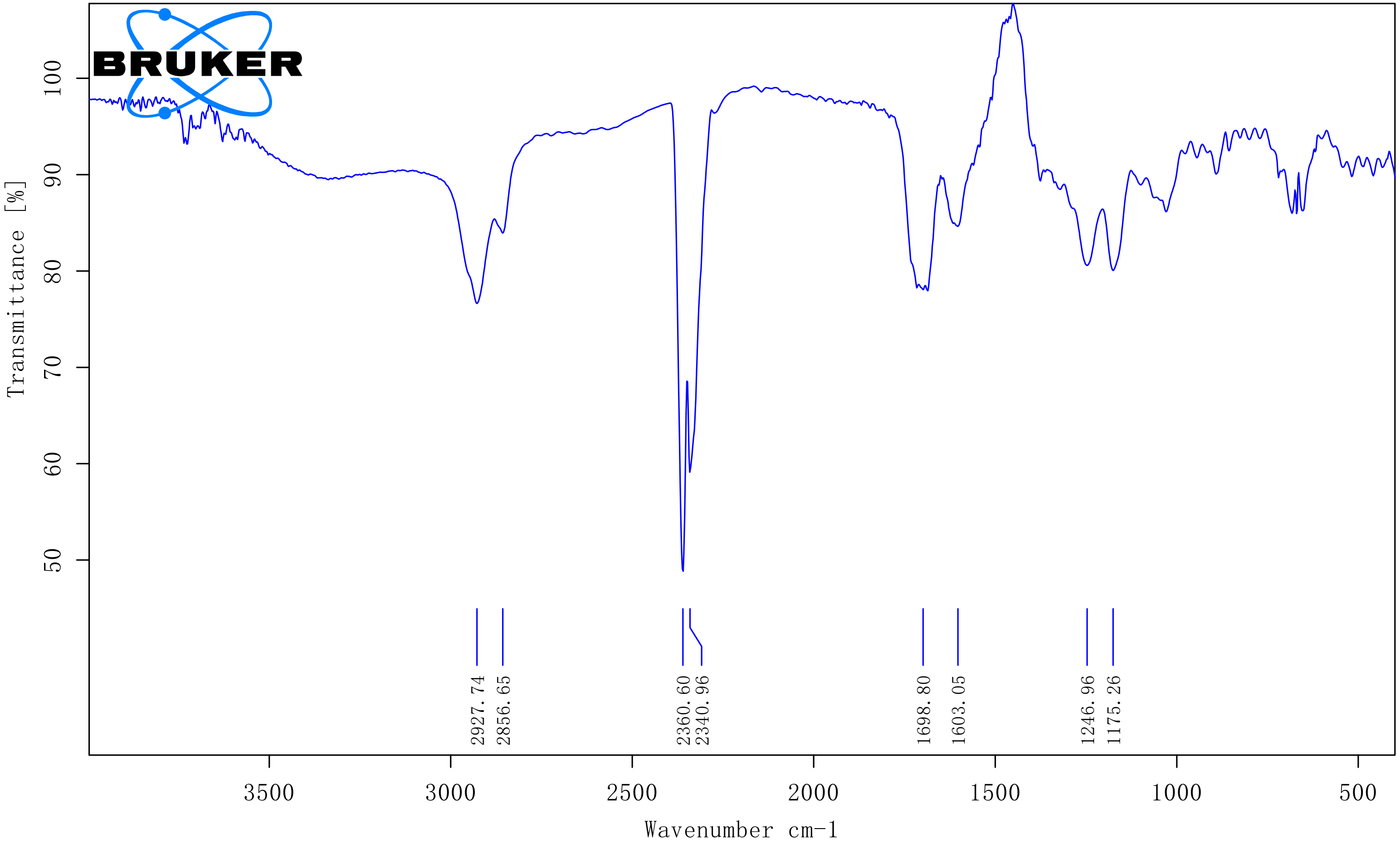


| | | | |
|---|---|---|---|
| D:\My Documents\Bruker\OPUS_7.5.18\DATA\MEAS\GO-3.24 | GO-3 | Instrument type and / or accessory | 2018-8-18 |



Figure S 19. IR spectrum of compound **3**

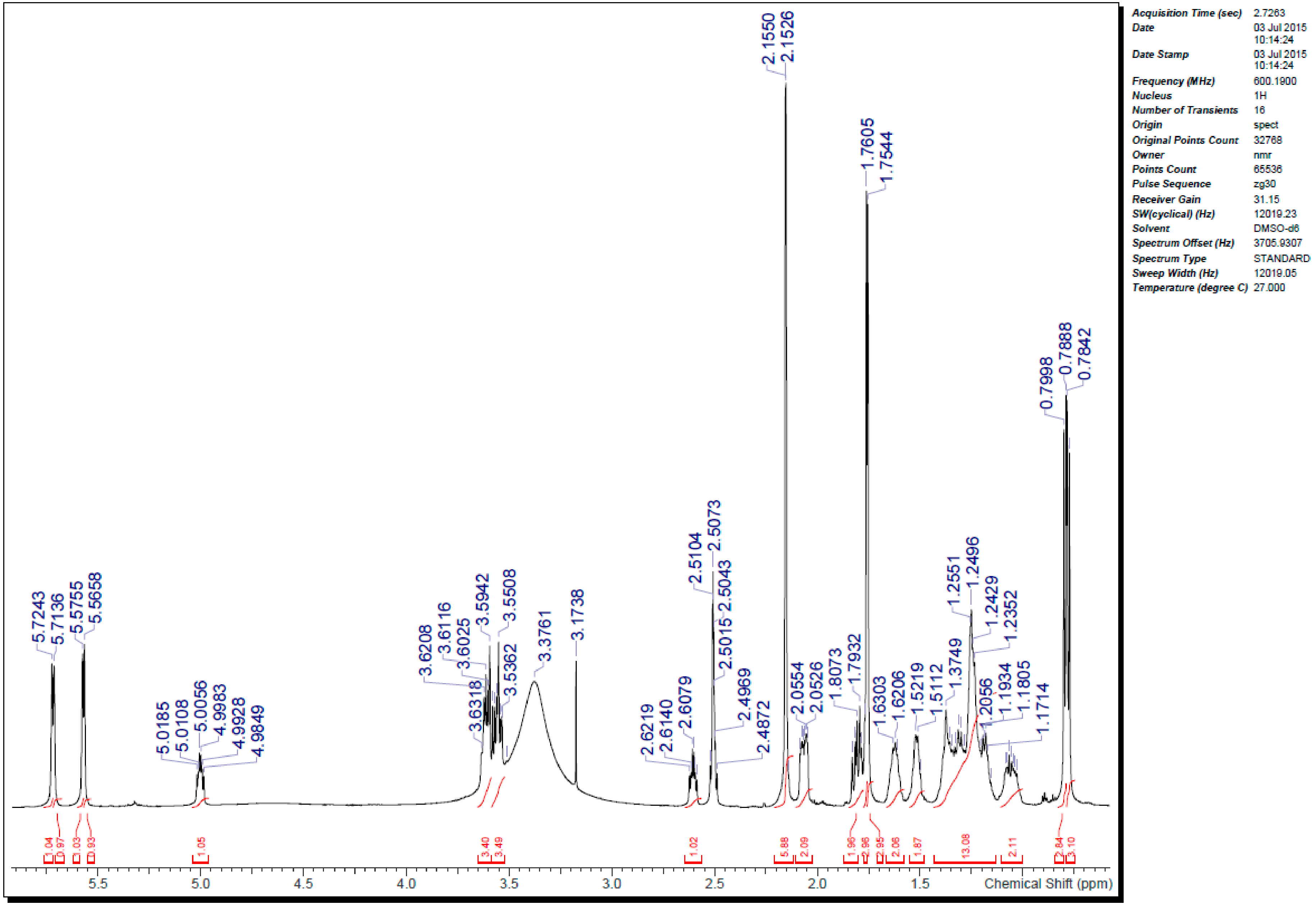


Figure S 20. $^{1}$H-NMR (600 MHz, DMSO-$d_6$) of compound **3**

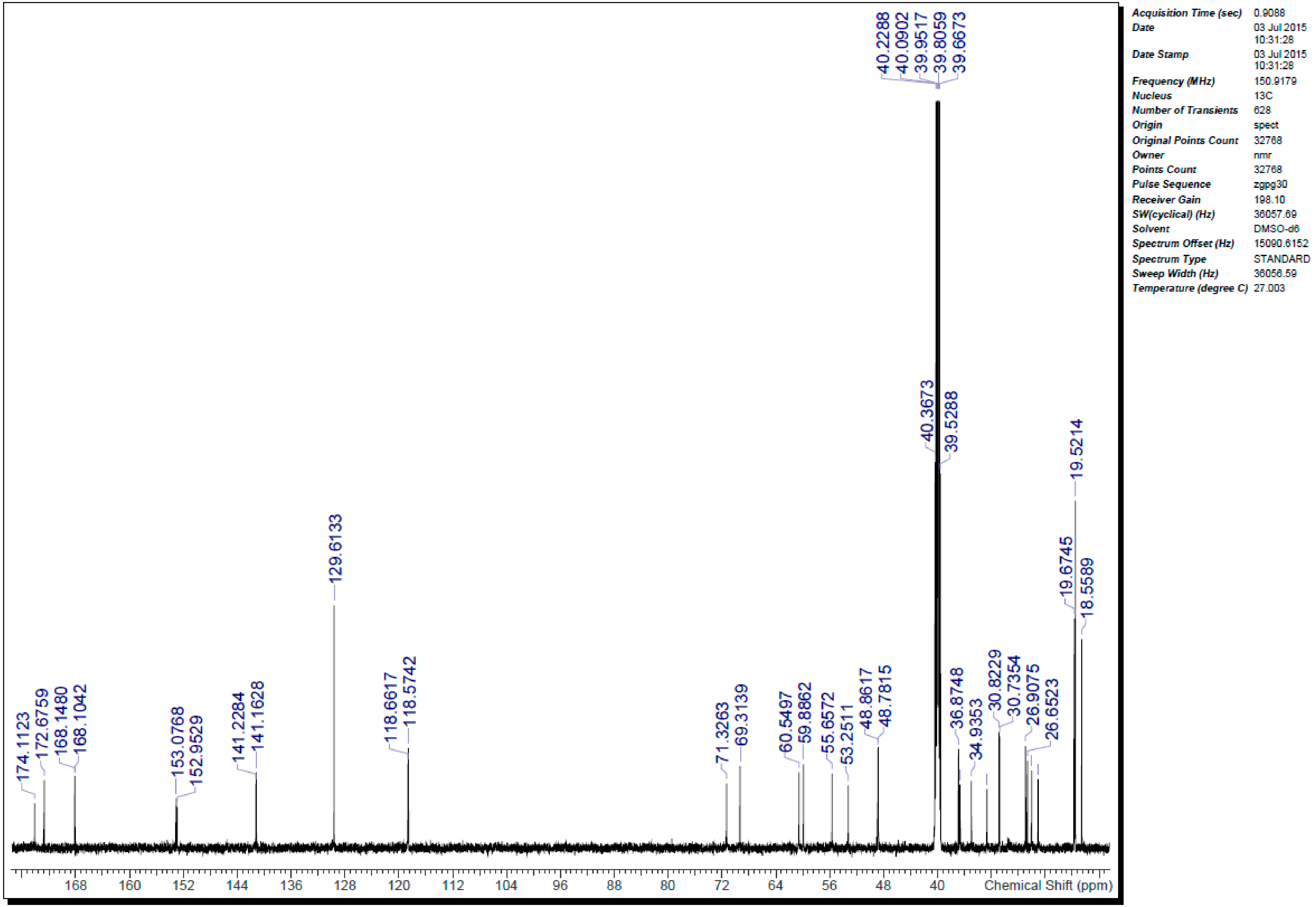


Figure S 21. $^{13}$C-NMR (150 MHz, DMSO-$d_6$) of compound **3**

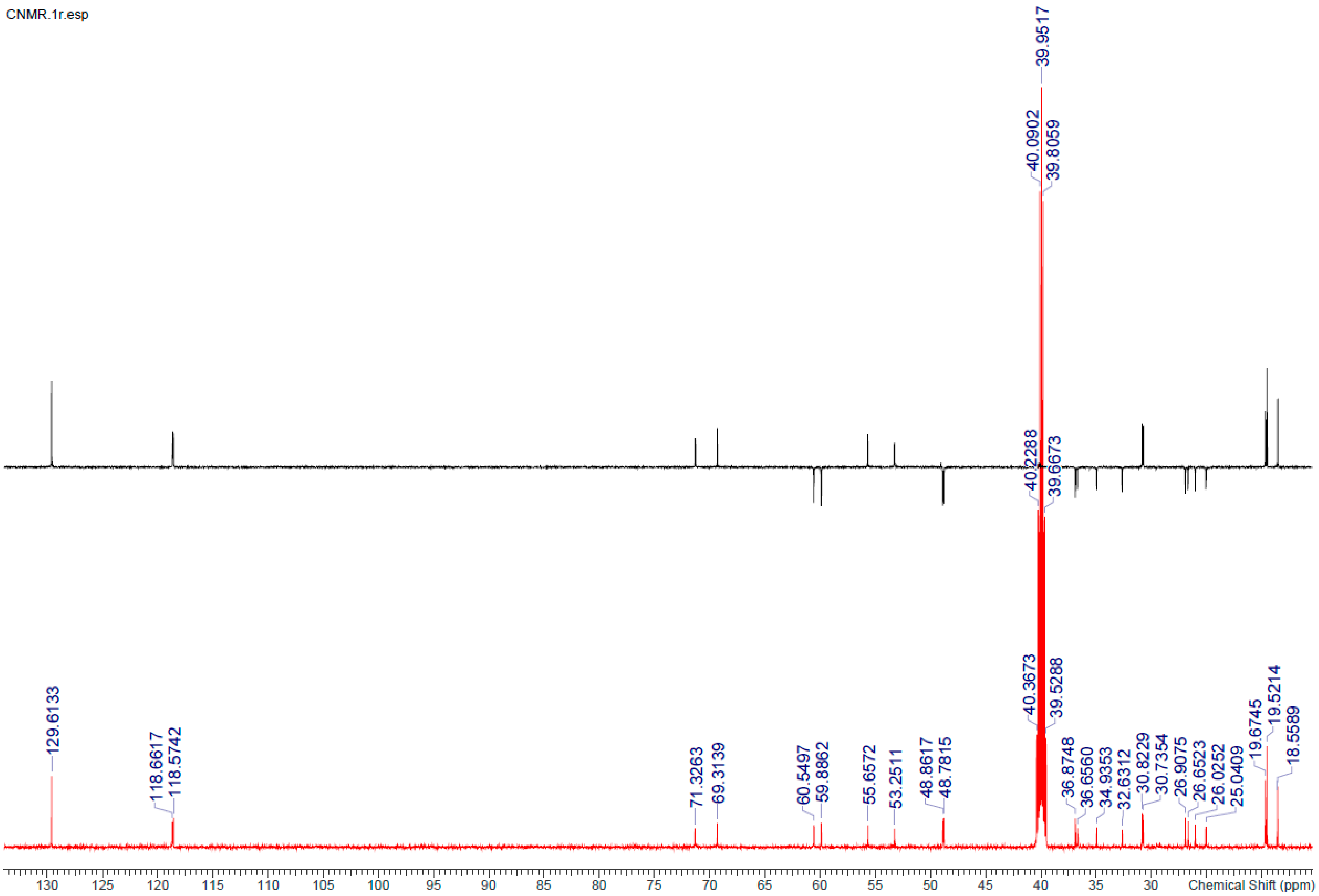


Figure S 22. DEPT spectrum of compound **3**

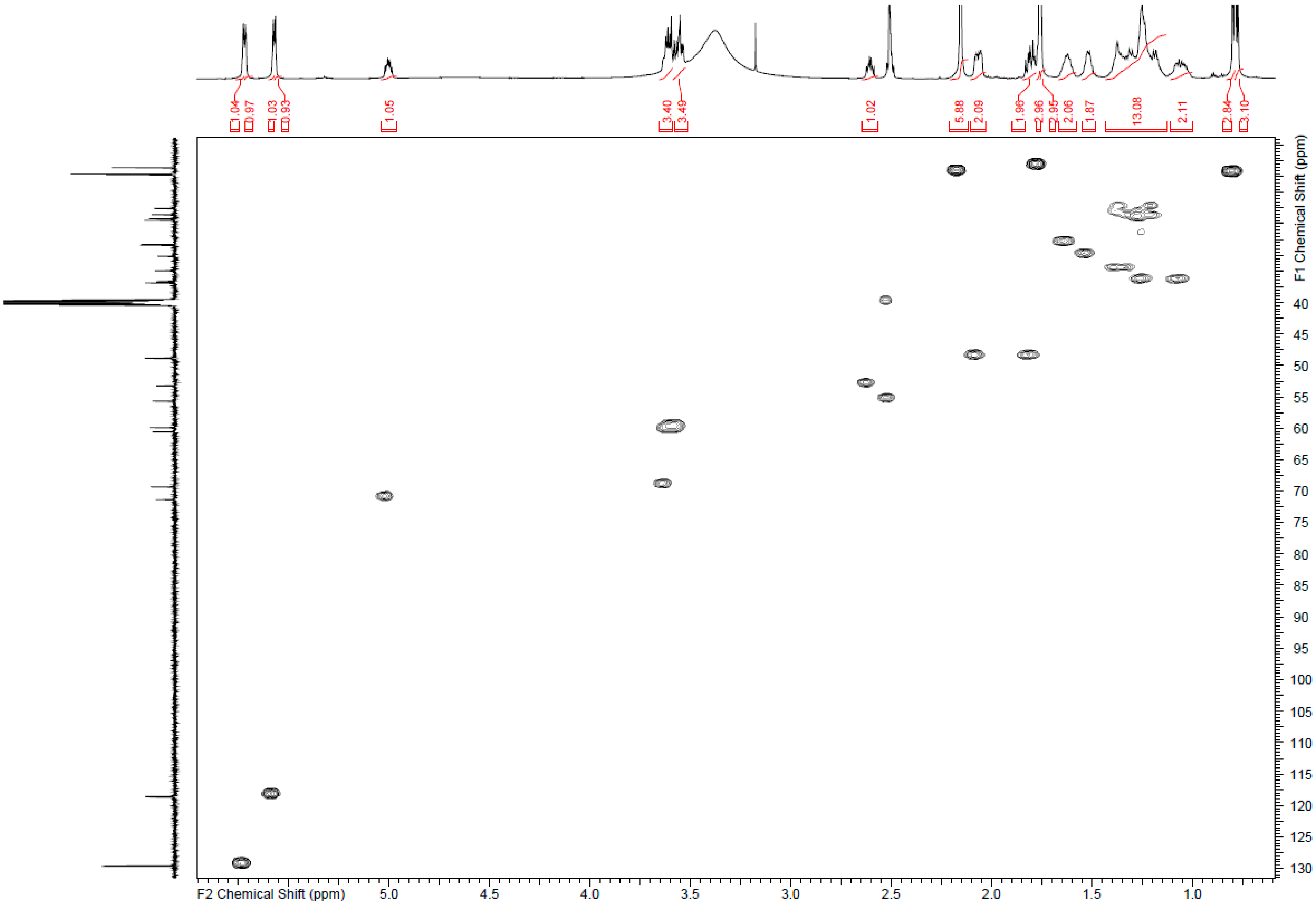


Figure S 23. HSQC spectrum of compound **3**

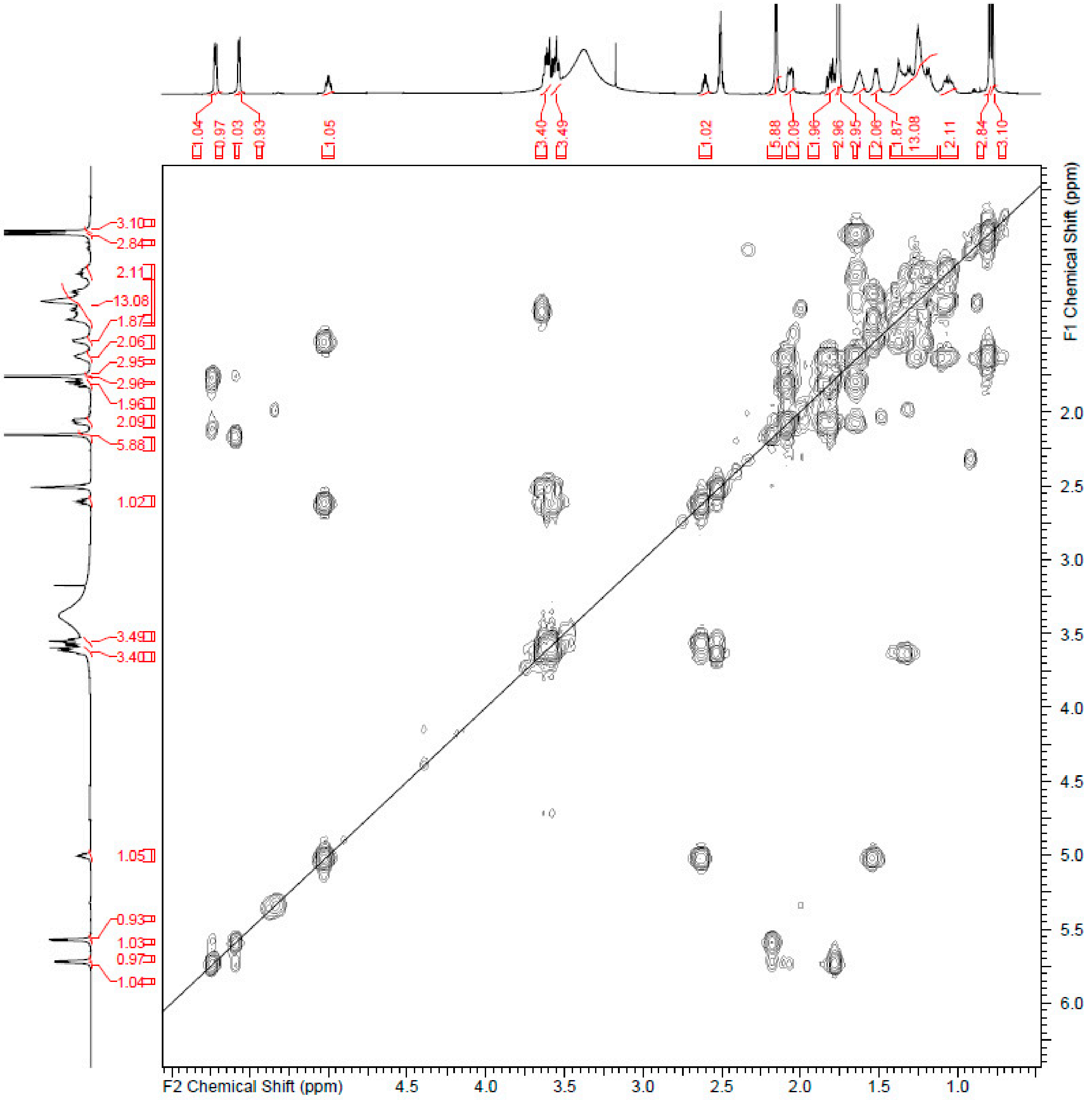

| | |
|---|---|
| *Acquisition Time (sec)* | (0.0852, 0.0214) |
| *Comment* | 5 mm PABBO BB/19F-1H/D Z-GRD Z114607/0133 |
| *Date* | 24 Nov 2016 21:39:28 |
| *File Name* | C:\Users\Administrator\Desktop\yanxia-20161125\H915CM5-1-90%-2\3\pdata\1\2rr |
| *Frequency (MHz)* | (600.1936, 600.1936) |
| *Nucleus* | (1H, 1H) |
| *Number of Transients* | 4 |
| *Origin* | spect |
| *Original Points Count* | (1024, 256) |
| *Owner* | nmr |
| *Points Count* | (2048, 1024) |
| *Pulse Sequence* | cosygpmfqf |
| *Solvent* | DMSO-d6 |
| *Spectrum Type* | COSY |
| *Sweep Width (Hz)* | (12013.36, 11978.70) |
| *Temperature (degree C)* | 23.963 |
| *Title* | |

Figure S 24. $^{1}H$-$^{1}H$ COSY spectrum of compound **3**

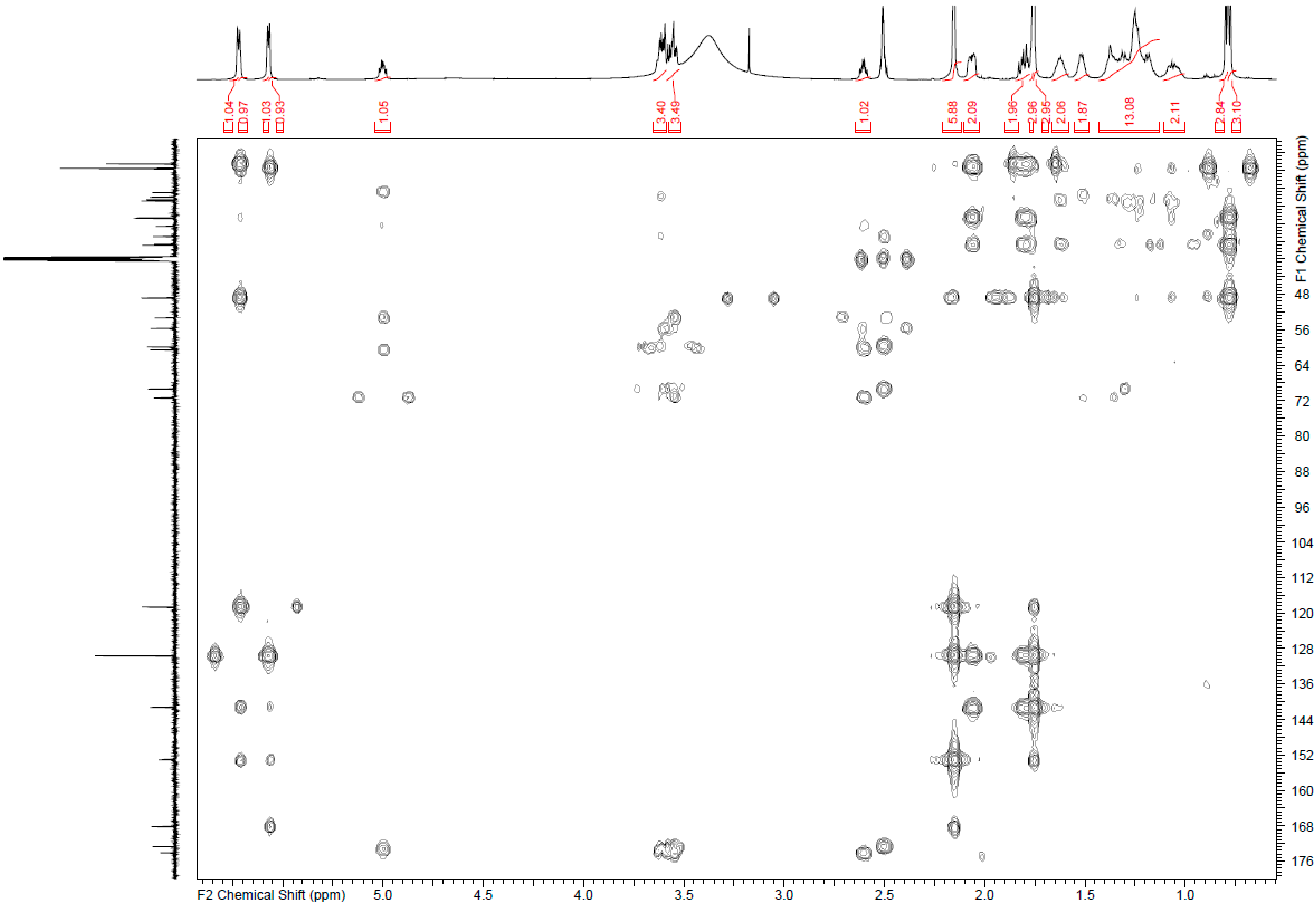


Figure S 25. HMBC spectrum of compound **3**

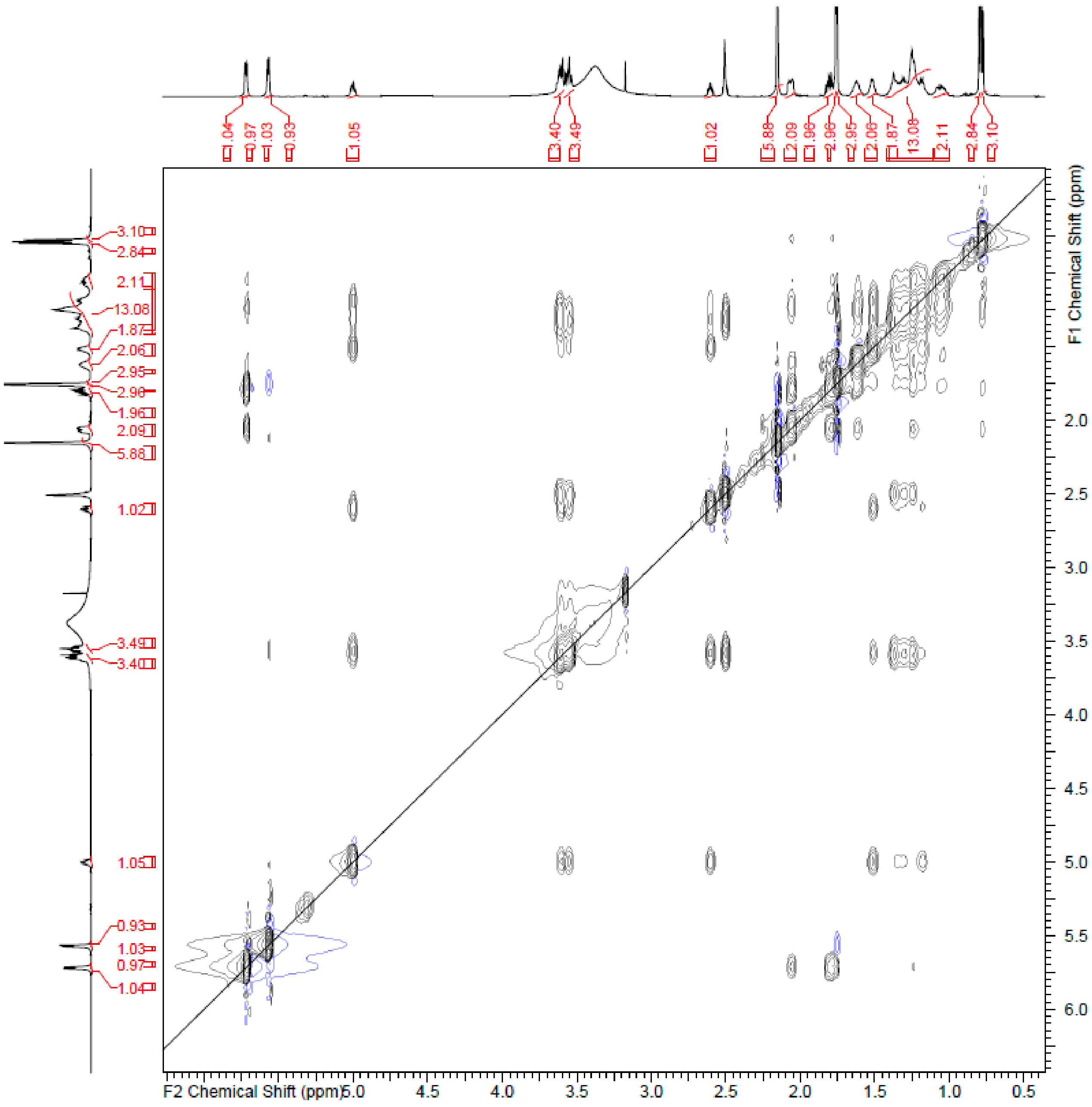


| | |
|---|---|
| *Acquisition Time (sec)* | (0.1167, 0.0292) |
| *Comment* | 5 mm PABBO BB/19F-1H/D Z-GRD Z114607/0133 |
| *Date* | 25 Nov 2016 08:59:28 |
| *File Name* | C:\Users\Administrator\Desktop\yanxia-20 161125\H915CM5-1-90%-2\6\pdata\1\2rr |
| *Frequency (MHz)* | (600.1937, 600.1937) |
| *Nucleus* | (1H, 1H) |
| *Number of Transients* | 4 |
| *Origin* | spect |
| *Original Points Count* | (1024, 256) |
| *Owner* | nmr |
| *Points Count* | (1024, 1024) |
| *Pulse Sequence* | noesygpphpp |
| *Solvent* | DMSO-d6 |
| *Spectrum Type* | NOESY |
| *Sweep Width (Hz)* | (8763.36, 8763.36) |
| *Temperature (degree C)* | 24.001 |
| *Title* | |

Figure S 26. NOESY spectrum of compound **3**

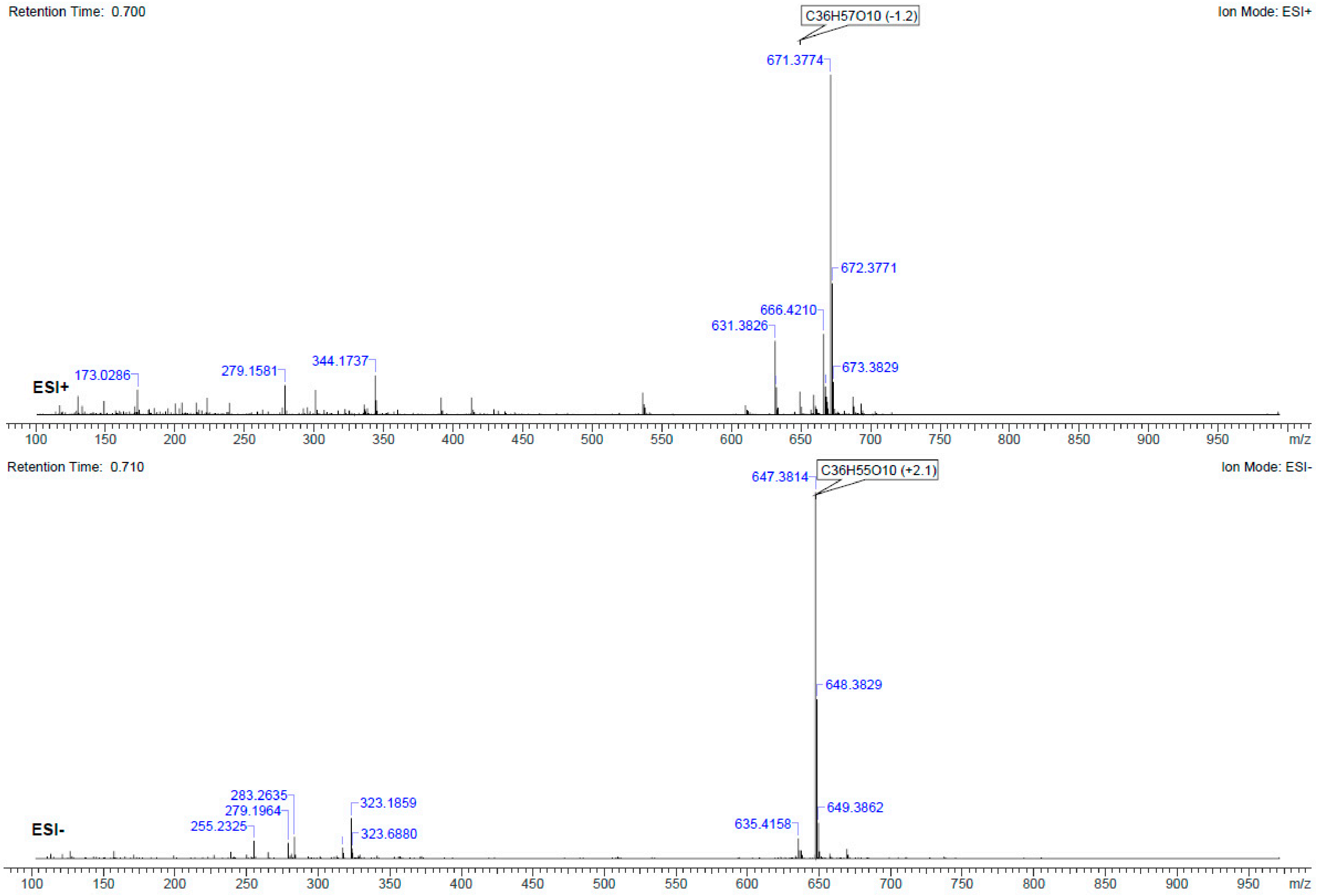


Figure S 27. HR-ESI-MS spectra of compound **4**

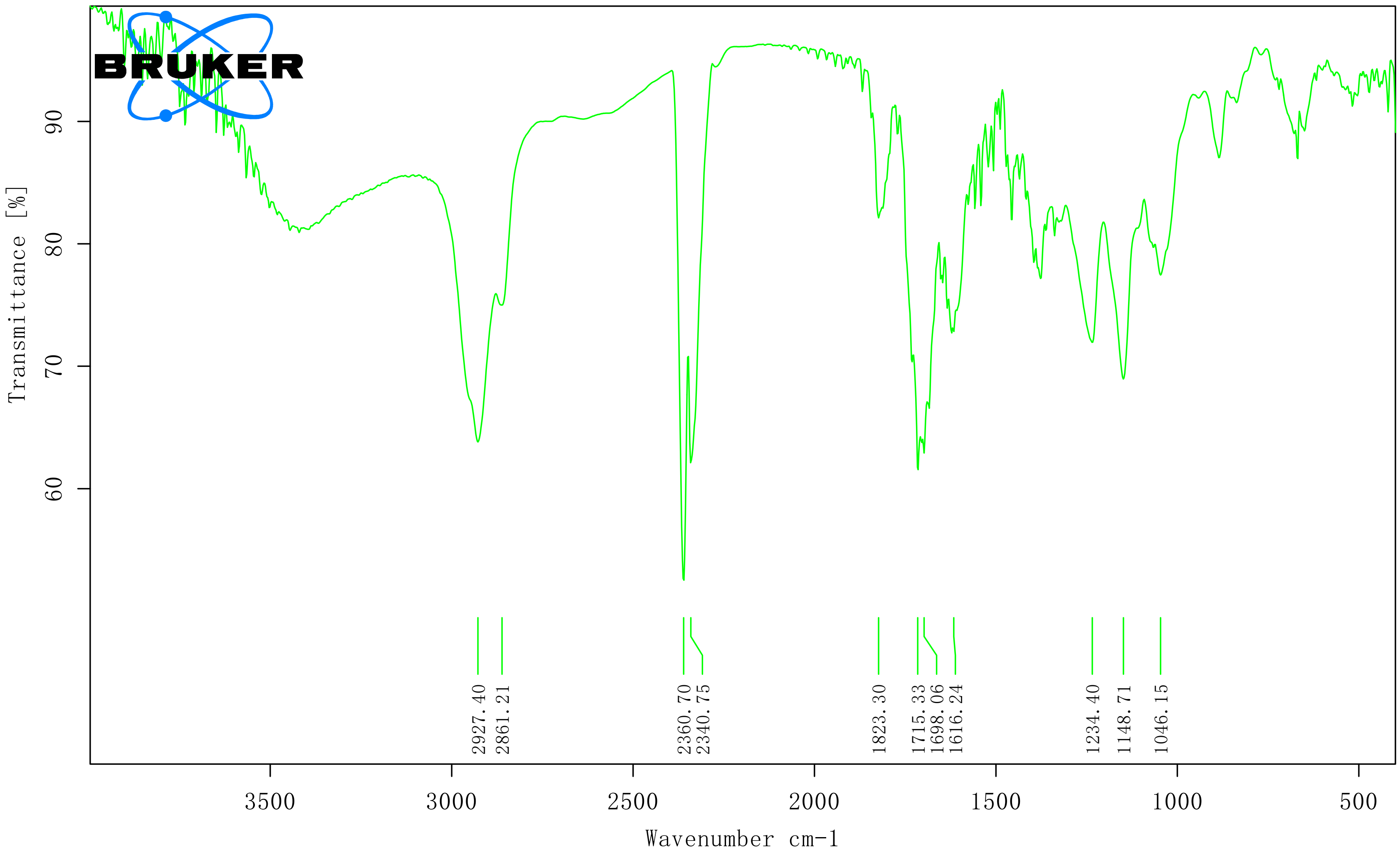


| | | | |
|---|---|---|---|
| D:\My Documents\Bruker\OPUS_7.5.18\DATA\MEAS\GO-3.28 | GO-3 | Instrument type and / or accessory | 2018-8-20 |



Figure S 28. IR spectrum of compound **4**

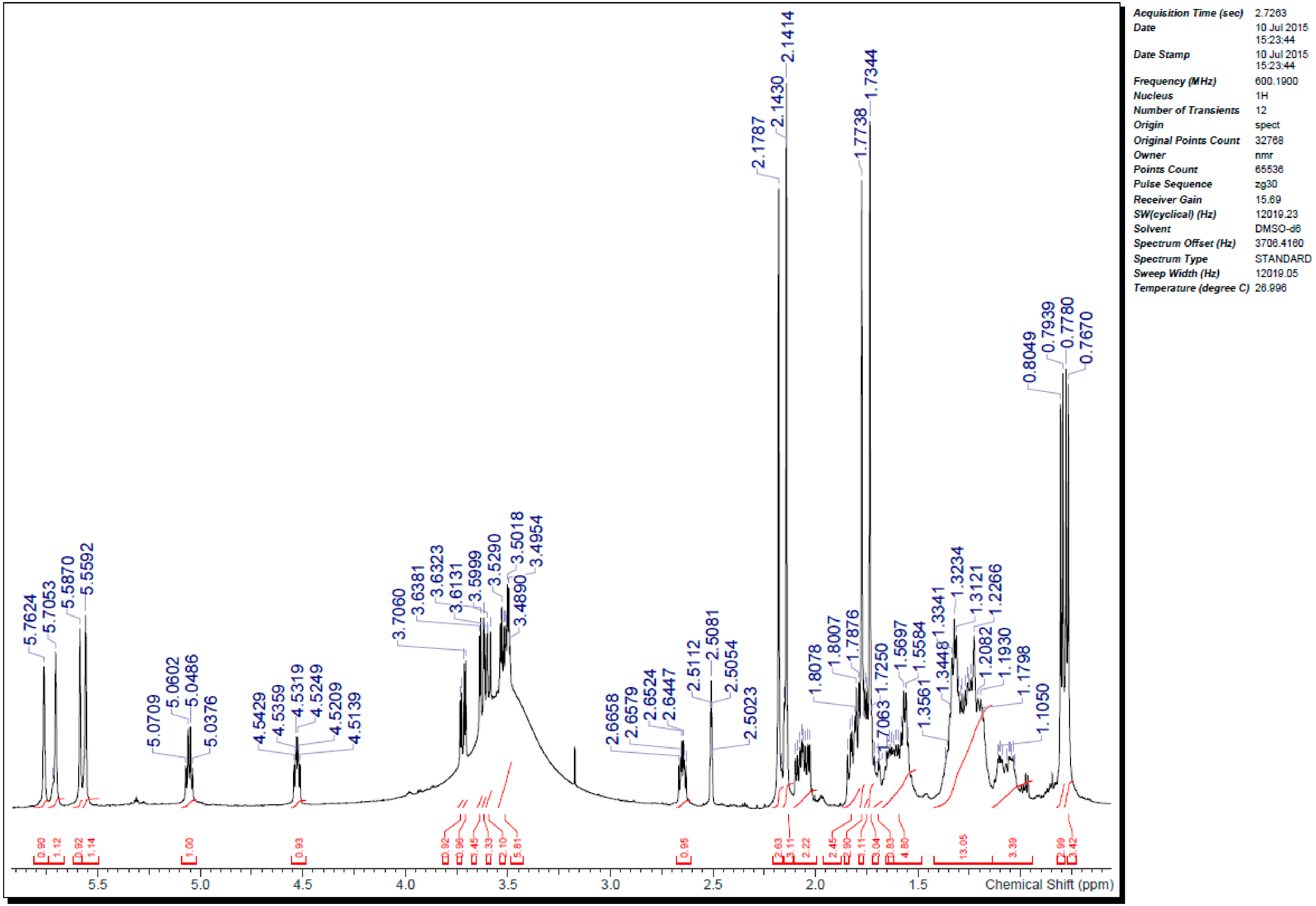


Figure S 29. $^{1}$H-NMR (600 MHz, DMSO-$d_6$) of compound **4**

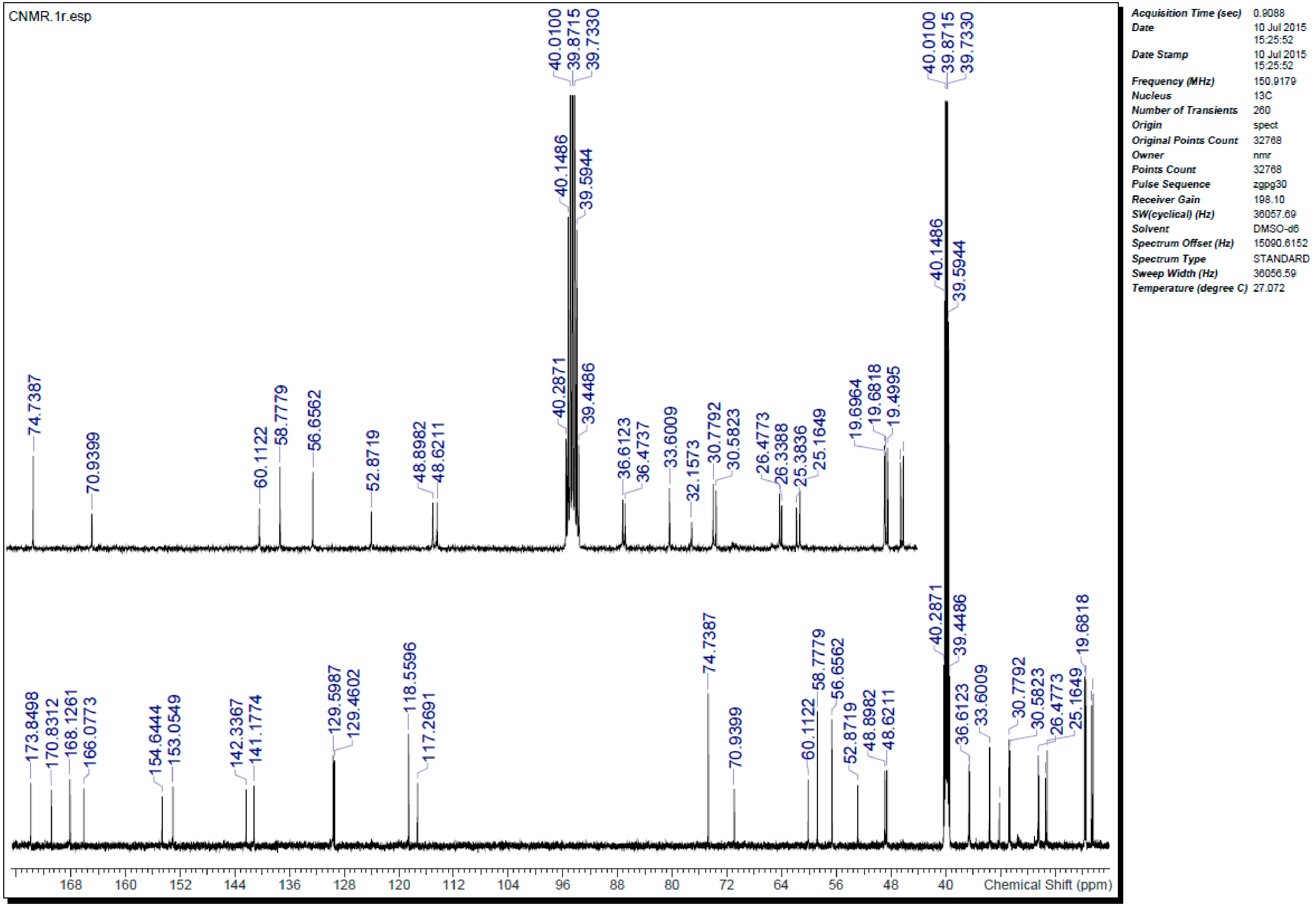


Figure S 30. $^{13}$C-NMR (150 MHz, DMSO-$d_6$) of compound **4**

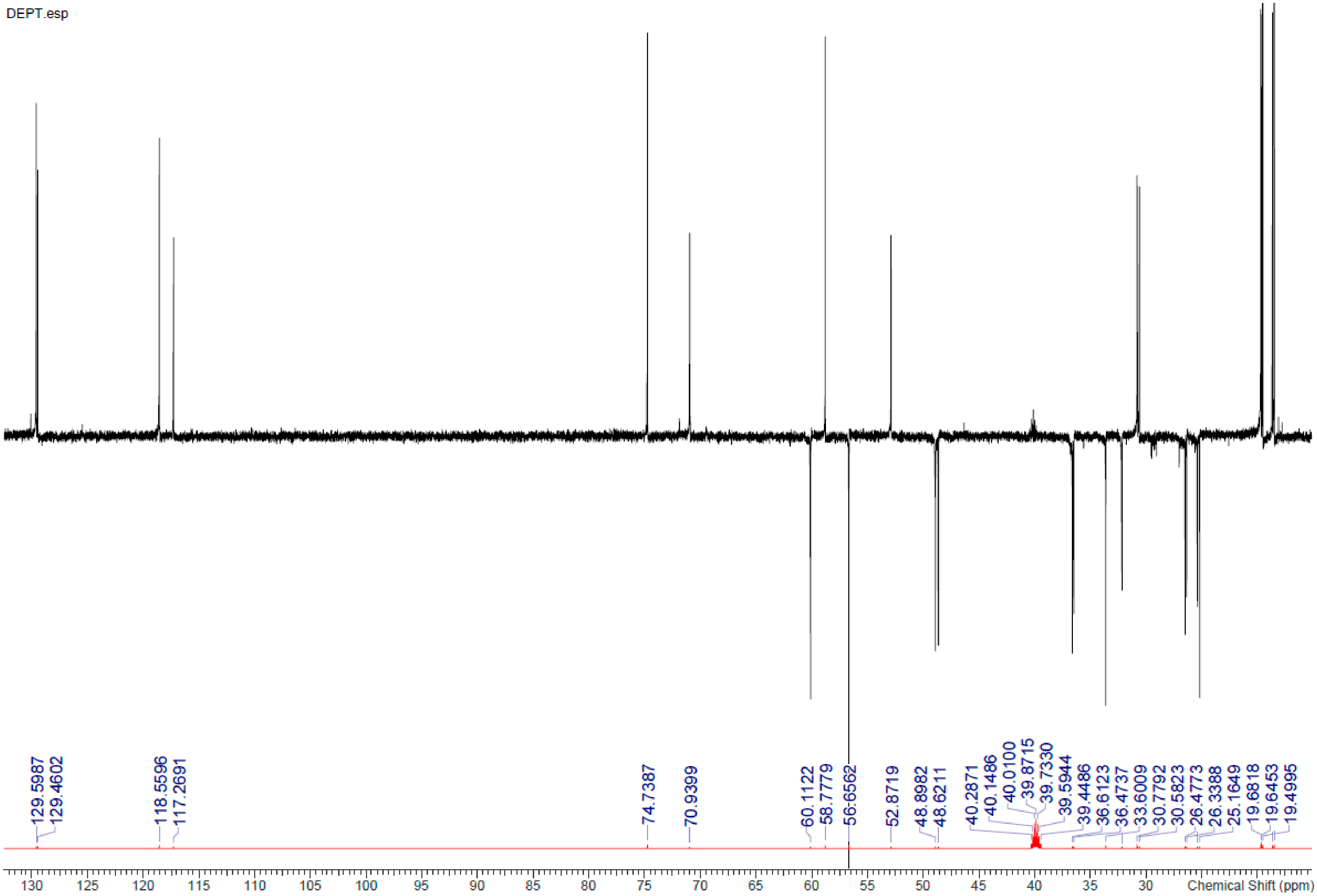


Figure S 31. DEPT spectrum of compound **4**

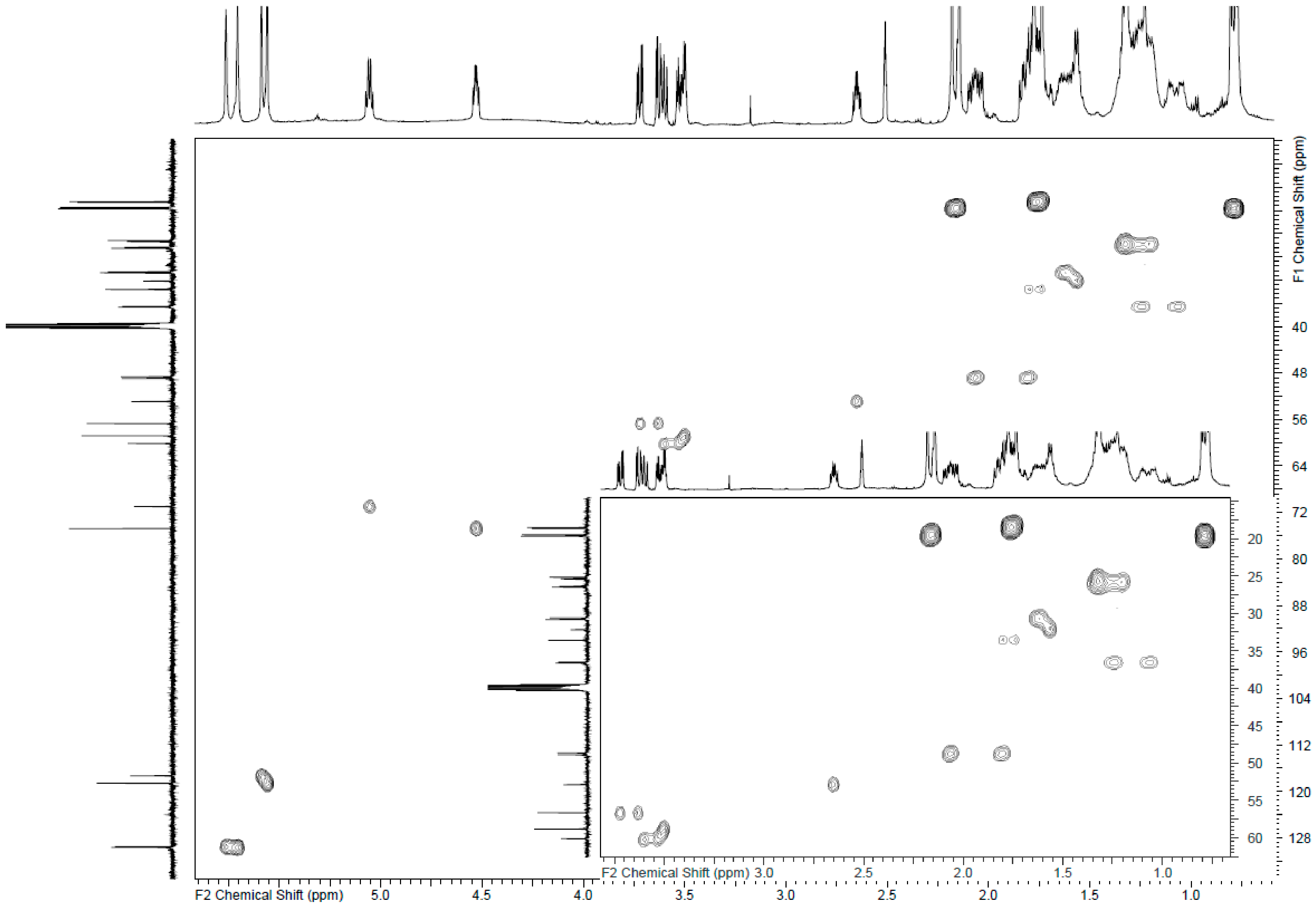


Figure S 32. HSQC spectrum of compound **4**

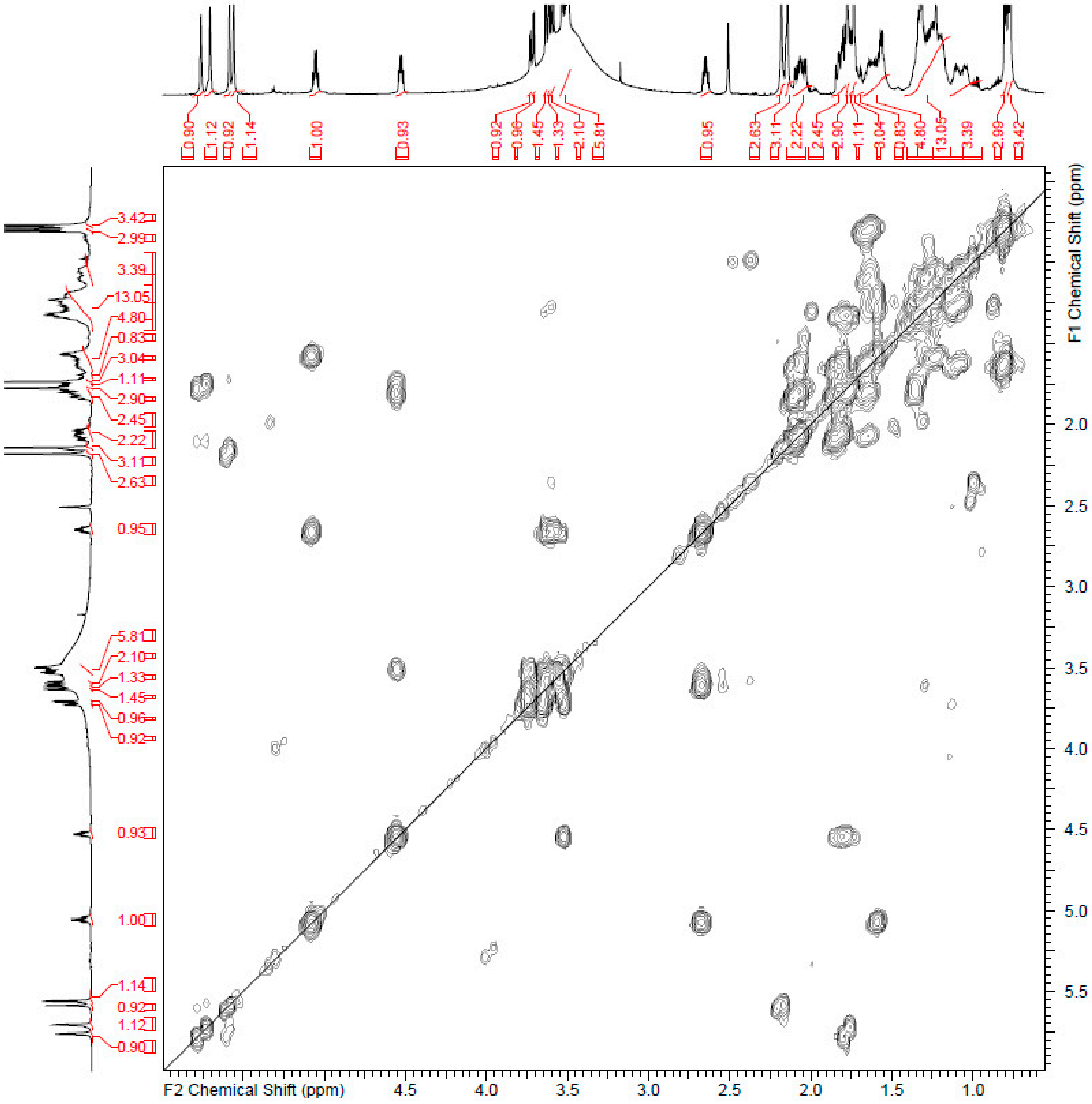


| | |
|---|---|
| *Acquisition Time (sec)* | (0.0852, 0.0214) |
| *Comment* | 5 mm PABBO BB/19F-1H/D Z-GRD Z114607/0133 |
| *Date* | 28 Sep 2015 14:25:04 |
| *File Name* | C:\Users\Administrator\Desktop\yanxia-20150929\H915CM10-1-90%-1\3\pdata\1\2rr |
| *Frequency (MHz)* | (600.1936, 600.1936) |
| *Nucleus* | (1H, 1H) |
| *Number of Transients* | 4 |
| *Origin* | spect |
| *Original Points Count* | (1024, 256) |
| *Owner* | nmr |
| *Points Count* | (2048, 1024) |
| *Pulse Sequence* | cosygpmfqf |
| *Solvent* | DMSO-d6 |
| *Spectrum Type* | COSY |
| *Sweep Width (Hz)* | (12013.36, 11978.70) |
| *Temperature (degree C)* | 26.945 |
| *Title* | |

Figure S 33. $^{1}H$-$^{1}H$ COSY spectrum of compound **4**

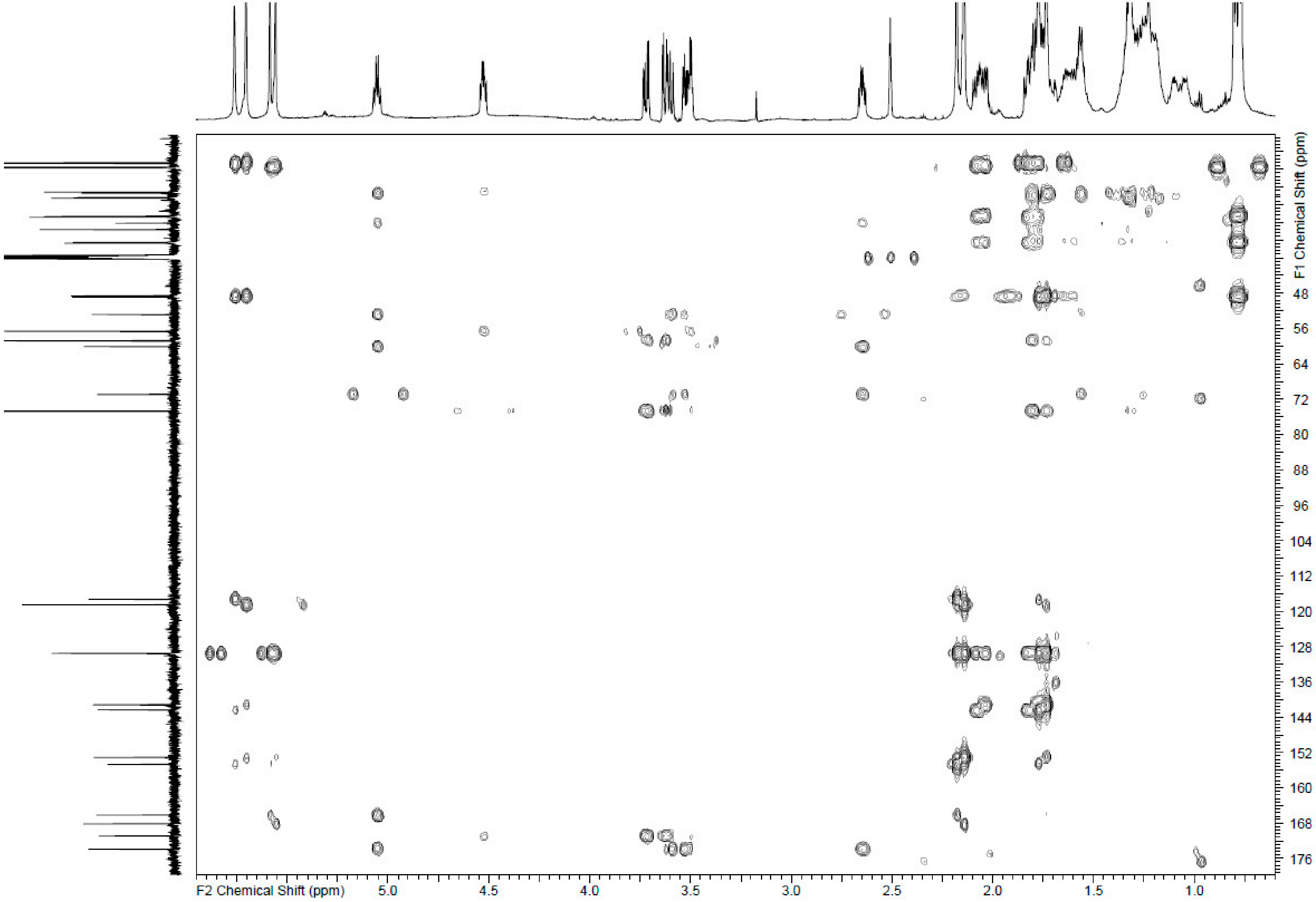


Figure S 34. HMBC spectrum of compound **4**

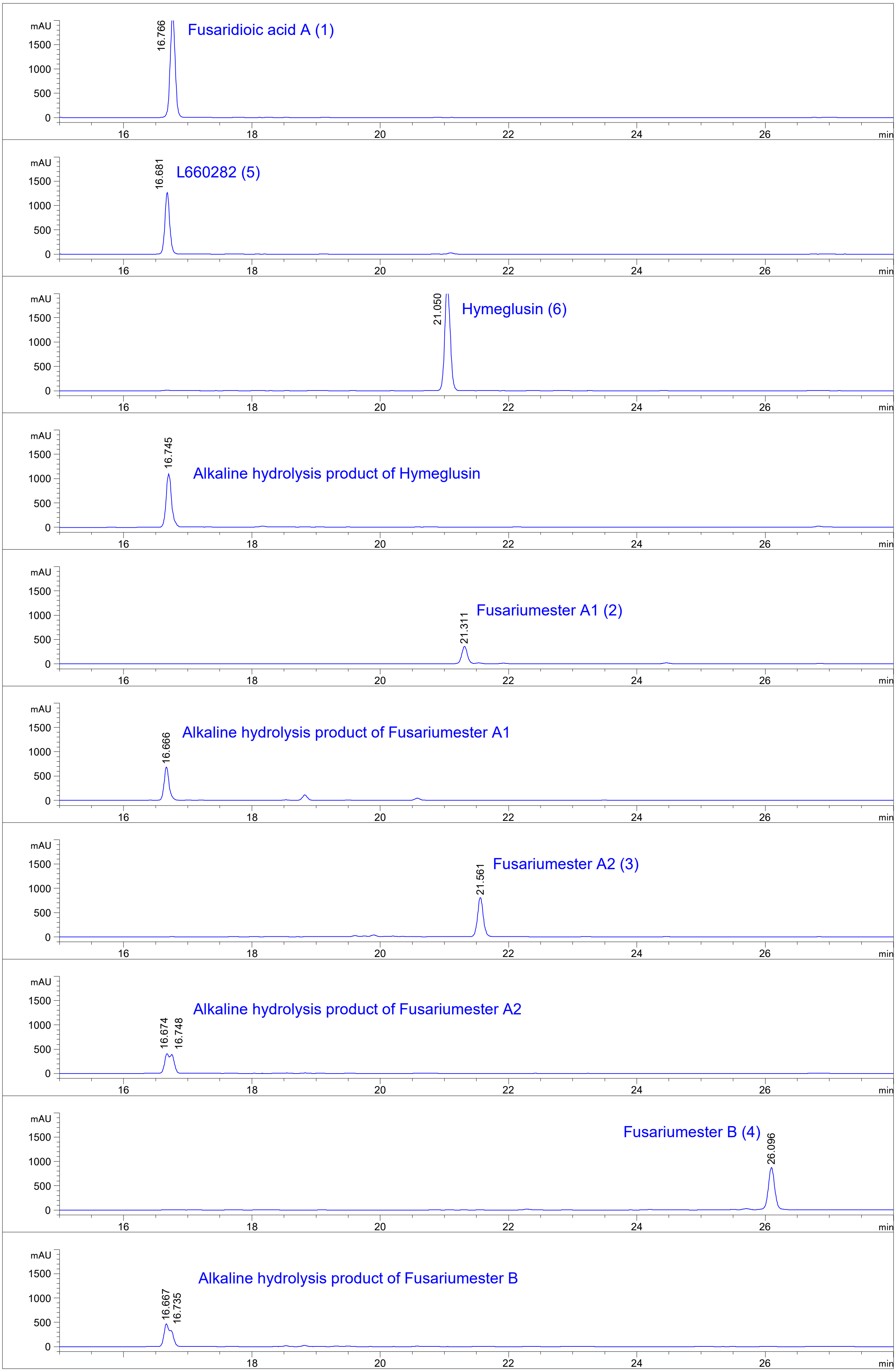


Figure S 35. HPLC chromatograms of compounds **1-6** and alkaline hydrolysis products of **2-4**, **6**
